# Lysosomal EGFR acts as a Rheb-GEF independent of its kinase activity to activate mTORC1

**DOI:** 10.1038/s41422-025-01110-x

**Published:** 2025-04-21

**Authors:** Xiaobo He, Qiu-Xia Wang, Denghui Wei, Yujie Lin, Xia Zhang, Yuanzhong Wu, Xuexia Qian, Zhihao Lin, Beibei Xiao, Qinxue Wu, Zhen Wang, Fengtao Zhou, Zhihao Wei, Jingxuan Wang, Run Gong, Ruhua Zhang, Qingling Zhang, Ke Ding, Song Gao, Tiebang Kang

**Affiliations:** 1 Sun Yat-sen University Cancer Center, Guangdong Provincial Clinical Research Center for Cancer, State Key Laboratory of Oncology in South China, Guangzhou, Guangdong China; 2 Department of Pathology, Guangdong Provincial People’s Hospital (Guangdong Academy of Medical Sciences), Southern Medical University, Guangzhou, Guangdong China; 3 State Key Laboratory of Chemical Biology, Shanghai Institute of Organic Chemistry, Chinese Academy of Sciences, Shanghai, China; 4 International Cooperative Laboratory of Traditional Chinese Medicine Modernization and Innovative Drug Development, Ministry of Education (MoE) of People’s Republic of China, College of Pharmacy, Jinan University, Guangzhou, Guangdong China; 5 Department of Oncology Radiotherapy, The First Affiliated Hospital, Hengyang Medical School, University of South China, Hengyang, Hunan China; 6 Integrated Traditional Chinese and Western Medicine Research Center, Sun Yat-sen University Cancer Center, Guangzhou, Guangdong China

**Keywords:** TOR signalling, Non-small-cell lung cancer

## Abstract

Oncogenic mutations in EGFR often result in EGF-independent constitutive activation and aberrant trafficking and are associated with several human malignancies, including non-small cell lung cancer. A major consequence of EGFR mutations is the activation of the mechanistic target of rapamycin complex 1 (mTORC1), which requires EGFR kinase activity and downstream PI3K/AKT signaling, resulting in increased cell proliferation. However, recent studies have elucidated kinase-independent roles of EGFR in cell survival and cancer progression. Here, we report a *cis* mTORC1 activation function of EGFR that is independent of its kinase activity. Our results reveal that lysosomal localization of EGFR is critical to mTORC1 activation, where EGFR physically binds Rheb, acting as a guanine exchange factor (GEF) for Rheb, with its Glu804 serving as a potential glutamic finger. Genetic knock-in of EGFR-E804K in cells reduces the level of GTP-bound Rheb, and significantly suppresses mTORC1 activation, cell proliferation and tumor growth. Different tyrosine kinase inhibitors exhibit distinct effects on EGFR-induced mTORC1 activation, with afatinib, which additionally blocks EGFR’s GEF activity, causing a much greater suppression of mTORC1 activation and cell growth, and erlotinib, which targets only kinase activity, resulting in only a slight decrease. Moreover, a novel small molecule, BIEGi-1, was designed to target both the Rheb-GEF and kinase activities of EGFR, and shows a strong inhibitory effect on the viability of cells harboring EGFR mutants. These findings unveil a fundamental event in cell growth and suggest a promising strategy against cancers with EGFR mutations.

## Introduction

The mechanistic target of rapamycin complex 1 (mTORC1) is a hub for cell growth and metabolism by modulating the synthesis of proteins, lipids, and nucleotides, as well as autophagy.^1–4^ Activation of mTORC1 is dependent on two distinct small GTPases, Rag and Rheb, located on the lysosomal surface.^5–7^ Rag GTPases are responsible for the translocation of mTORC1 to the lysosome upon the sensing of amino acids by cells,^5,8–10^ where the direct interaction with GTP-bound Rheb leads to mTORC1 activation.^6,11^ This Rheb-mTORC1 crosstalk is orchestrated by upstream signals, including growth factors, cellular energy status, and stress responses.^12,13^ As indicated by previous studies, these signals converge at tuberous sclerosis complex 2 (TSC2), a specific GTPase-activating protein (GAP), that renders Rheb to the inhibitory GDP-bound state for mTORC1.^14–18^

Being key upstream signaling molecules of Rheb, receptor tyrosine kinases (RTKs), including epidermal growth factor receptor (EGFR), play pivotal roles in modulating mTORC1 function.^19–22^ Activation of EGFR through ligand-induced dimerization leads to autophosphorylation and the initiation of a downstream signaling cascade via the phosphoinositide 3-kinase (PI3K)/AKT pathway.^23^ Activated AKT directly phosphorylates TSC2 and suppresses its Rheb-GAP function, which in turn favors GTP-bound Rheb and facilitates the activation of mTORC1.^24–26^ However, recent studies have elucidated kinase-independent roles of EGFR in cell survival and cancer progression,^27–29^ implicating EGFR in the activation of mTORC1 through yet unidentified kinase-independent mechanisms that are essential for cell survival.

Apart from the kinase activity, intracellular trafficking of EGFR is a meticulously regulated process that is critical for its functional role in cellular signaling.^20,30–32^ Upon activation, EGFR undergoes internalization predominantly via clathrin-mediated endocytosis.^33,34^ Once internalized, EGFR is sorted into early endosomes, where it can be either recycled to the plasma membrane for maintaining cell surface receptor levels, or targeted to lysosomes for degradation, claiming the termination of signaling.^30,35^ This dynamic regulation of EGFR transport and degradation is critical for controlling the intensity and duration of signaling pathways.^35^

Abnormal EGFR signaling, caused by amplification, overexpression, and mutations (particularly in the intracellular tyrosine kinase domain (TKD)), contributes to the development of various pathologies, including cancer.^36–38^ A prominent example is the link between EGFR mutations and the occurrence of non-small cell lung cancer (NSCLC).^36^ These oncogenic mutations often result in EGF-independent constitutive activation and aberrant trafficking.^39^ Previous evidence indicates that these mutant forms of EGFR colocalize with markers for both early/recycling endosomes and late endosomal/lysosomal compartments, indicating their presence in multiple endosomal compartments within the cell.^40^ EGFR tyrosine kinase inhibitors (EGFR-TKIs) are commonly used to treat NSCLC, mainly by blocking its kinase activity.^38,41^ However, inhibition of EGFR’s kinase activity alone may not be sufficient for effective tumor eradication.^42,43^ Therefore, the mechanisms by which aberrant endocytosed EGFR sustains cell survival are not well understood.

In this study, we analyzed lung adenocarcinoma specimens harboring EGFR mutations from patients who responded to first- or second-generation EGFR-TKI monotherapy. We discovered a new role of EGFR as a guanine exchange factor (GEF) for Rheb independent of its kinase activity. Restricting the Rheb-GEF function is an effective strategy to suppress the growth of cancer cells harboring oncogenic EGFR mutations.

## Results

### Different EGFR-TKIs have distinct impacts on mTORC1 activation

**Fig. 1:**
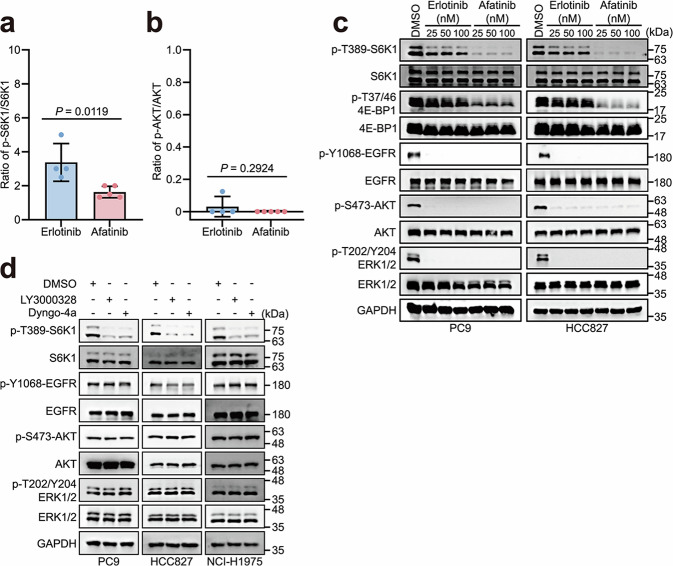
Lysosomal EGFR is crucial for mTORC1 activation. **a**, **b** Quantification of the p-T389-S6K1/S6K1 (**a**) and p-S473-AKT/AKT (**b**) ratios in NSCLC patients tested by IHC. Data are presented as means ± SD. Two-tailed unpaired *t*-test. **c** Afatinib causes much stronger inhibition of mTORC1 activation than erlotinib, although both are capable of suppressing EGFR tyrosine kinase activity. PC9 and HCC827 cells were treated with erlotinib or afatinib at the indicated dose for 12 h and analyzed by western blotting. **d** LY3000328 or Dyngo-4a impairs the activation of mTORC1 in cells harboring mutant EGFR. PC9, HCC827, or NCI-H1975 cells were treated with DMSO, 50 μM LY3000328 for 24 h, or 50 μM Dyngo-4a for 2 h, and analyzed by western blotting.

Two recent clinical trials, LUX-Lung 7^44^ and ARCHER-1050,^45^ have demonstrated that second-generation EGFR-TKIs significantly prolong progression-free survival in patients with EGFR-mutant NSCLC compared to first-generation inhibitors. This enhanced efficacy may be explained by their irreversible binding to a broader range of mutant forms, and/or their ability to inhibit additional members of the ErbB family.^46,47^ Despite these advances, the status of EGFR-mediated mTORC1 activation in response to these inhibitors remains unclear. To address this issue, we assessed the status of S6K1 Thr389 phosphorylation (p-T389-S6K1), an indicator of mTORC1 activity, in lung adenocarcinoma specimens from patients with EGFR mutations who were treated only with erlotinib (first-generation EGFR-TKI) or afatinib (second-generation EGFR-TKI) as neoadjuvant therapy (Supplementary information, Table S1). Immunohistochemistry (IHC) revealed that the ratios of p-T389-S6K1 to total S6K1 were much lower in the afatinib-treated group than those of the erlotinib-treated group (*P* = 0.0119; Fig. 1a; Supplementary information, Fig. S1), suggesting strong mTORC1-suppressing potential of afatinib. In contrast, both afatinib and erlotinib exhibited comparable inhibitory effects on AKT activation in these patients, as indicated by similar levels of AKT Ser473 phosphorylation between the two groups (*P* = 0.2924; Fig. 1b).

To verify these findings in cellular models, we treated PC9 and HCC827 cells, both harboring endogenous EGFR mutants, with these two EGFR-TKIs. Consistent with the findings in clinical samples, erlotinib-treated cells had substantially higher mTORC1 activation than those treated with afatinib, indicated by both p-T389-S6K1 and phosphorylation (Thr37/46) of 4E-Binding Protein 1 (p-T37/46-4E-BP1) (Fig. 1c). In the meantime, the levels of phosphorylated EGFR, AKT, and ERK1/2 in these cells were suppressed at comparable levels (Fig. 1c). These results suggest that the kinase activity of EGFR is not indispensable for mTORC1 activation.

### Lysosomal localization of EGFR is crucial for mTORC1 activation

Next, we sought to investigate how EGFR activates mTORC1 independent of its kinase activity. Oncogenic mutations in EGFR lead to aberrant trafficking, characterized by continuous ligand-independent internalization and subsequent lysosomal degradation.^40,48^ As mTORC1 activation occurs on the lysosomal surface,^3^ we examined whether inhibiting EGFR translocation to the lysosome suppresses mTORC1 activation using various translocation-blocking methods.^49^ The application of LY3000328, a cathepsin S inhibitor that disrupts endogenous mutant EGFR transfer from late endosomes to lysosomes, or Dyngo-4a, a dynamin inhibitor that impedes endocytosis of EGFR, resulted in dramatically reduced co-localization of EGFR with lysosomal-associated membrane protein 1 (LAMP1) (Supplementary information, Fig. S2). Furthermore, the treatment of LY3000328 or Dyngo-4a effectively impaired mTORC1 activation without altering the phosphorylation states of EGFR, AKT, or ERK1/2 (Fig. 1d).

Unlike oncogenic mutant EGFR, the activation and internalization of wild-type (WT) EGFR depend on ligand binding, which further triggers post-endocytic sorting.^32^ Under low-dose EGF stimulation, WT EGFR predominantly recycles back to the plasma membrane, whereas high-dose EGF stimulation enhances WT EGFR endocytosis, facilitating its translocation from early endosomes to late endosomes or lysosomes.^50^ Indeed, using multiple WT EGFR cells, including HeLa cells, human embryonic kidney-293T (HEK-293T) cells, and mouse embryonic fibroblasts (MEFs), we observed that EGF at a physiological concentration (1 ng/mL) induced only weak mTORC1 activation (Supplementary information, Fig. S3a). In contrast, optimized full activation of mTORC1 was achieved with EGF at 100 ng/mL for 30 min in these cells (Supplementary information, Fig. S3a, b).

To investigate whether lysosomal translocation of WT EGFR is required for mTORC1 activation, we again utilized the inhibitors LY3000328 and Dyngo-4a. Consistent with our findings in mutant EGFR cells (Fig. 1d), treatment with these inhibitors suppressed mTORC1 activation under high-dose (100 ng/mL) EGF stimulation in WT EGFR-expressing cells (Supplementary information, Fig. S3c). These results indicate that lysosomal localization of EGFR is crucial for mTORC1 activation.

### The TKD of EGFR directly binds Rheb

**Fig. 2:**
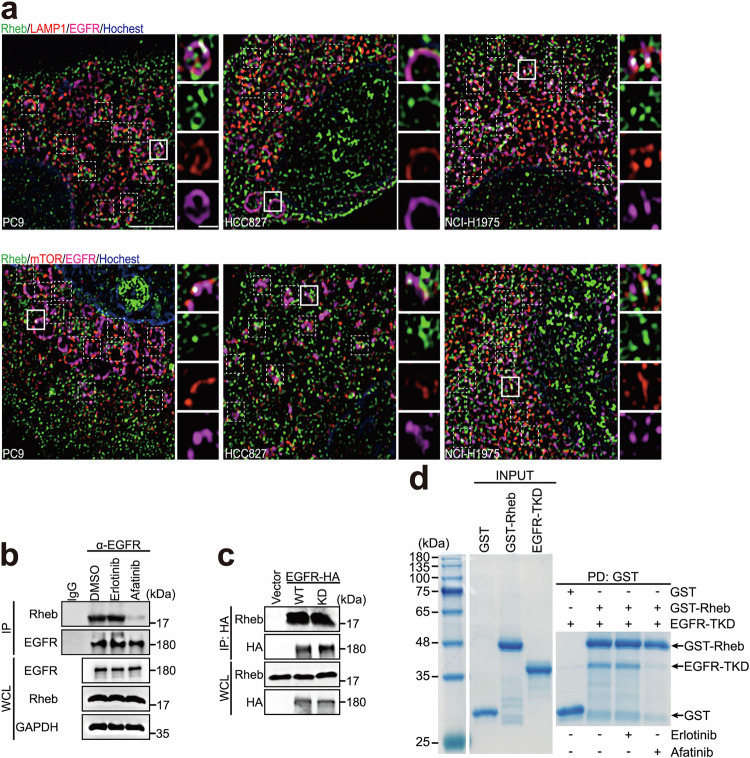
EGFR-TKD directly binds Rheb. **a** Co-localization of endogenous mutant EGFR with Rheb and LAMP1 (top) or mTOR (bottom). Immunofluorescence analysis of endogenous Rheb (green), EGFR (pink) and LAMP1 (top) or mTOR (bottom) (red) in EGFR-mutated cells using super-resolution SIM. Scale bar, 1 μm (enlarged view, 0.1 μm). **b** Afatinib inhibits the interaction between EGFR and Rheb in cells. PC9 cells treated with or without 25 nM erlotinib or afatinib for 12 h were subjected to immunoprecipitation and analyzed by western blotting. **c** The interaction between EGFR and Rheb is independent of EGFR kinase activity. HEK-293T cells stably expressing empty vector, HA-tagged EGFR WT or KD were subjected to immunoprecipitation and analyzed by western blotting. **d** Afatinib disrupts the EGFR–Rheb interaction in vitro. Purified EGFR-TKD was incubated with GST or GST-tagged Rheb_1–169_ (Rheb, unless specified) with or without 100 μM erlotinib or 100 μM afatinib, precipitated with GST beads, and subjected to SDS-PAGE analysis. Coomassie blue staining is shown.

Based on the requirement of lysosomal Rheb for mTORC1 activation,^6^ we further examined the cellular distributions of both EGFR and Rheb in cell lines harboring endogenous mutant EGFR, such as PC9, HCC827 and NCI-H1975. As shown in Supplementary information, Fig. S4a, a marked co-localization of endogenous mutant EGFR with Rheb around the nucleus was detected in these cells, a pattern that was hardly observed in cells expressing WT EGFR, such as HEK-293T and MEF. Using structure illumination microscopy (SIM), we found pronounced co-localization of endogenous mutant EGFR with Rheb and LAMP1, suggesting that mutant EGFR localizes to the lysosomal surface, where it interacts with Rheb (Fig. 2a, top). Additionally, the co-localization of mutant EGFR with Rheb and mTOR was further validated through SIM and lysosome immunoprecipitation (lyso-IP) (Fig. 2a, bottom; Supplementary information, Fig. S4b).

To understand whether this effect was also present in WT EGFR cells, we checked the co-localization of endogenous WT EGFR and Rheb by confocal microscopy during different time courses and at different concentrations of EGF stimulation in HeLa cells. Scattered co-localization of endocytosed EGFR and Rheb in the cytoplasm was observed (Supplementary information, Fig. S4c). At the optimized stimulation time of 30 min and a concentration of 100 ng/mL, a fluorescence overlay between endogenous WT EGFR and Rheb was clearly detected on lysosomes (as indicated by LAMP1-GFP; Supplementary information, Fig. S4d) but not on the endoplasmic reticulum (as indicated by Calnexin-mCherry) or Golgi apparatus (as indicated by mCherry-GM130; Supplementary information, Fig. S4e, f).

The interaction of EGFR with Rheb was confirmed by co-immunoprecipitation in cells (Fig. 2b), which was independent of EGFR tyrosine kinase activity, as both kinase-dead (KD) and WT EGFR had similar binding efficiencies for Rheb (Fig. 2c). Next, we examined the region of EGFR responsible for Rheb interaction. The TKD (amino acids 696–1022) of EGFR, but not the regulatory domain, immunoprecipitated with Rheb (Supplementary information, Fig. S5a, b). Additionally, purified EGFR-TKD could be physically pulled down by glutathione-S-transferase (GST)-tagged Rheb, which had a substantial GDP-bound fraction (Fig. 2d; Supplementary information, Fig. S5c). Notably, afatinib, but not erlotinib, disrupted the interaction of EGFR with Rheb both in cells and in vitro (Fig. 2b, d). These results demonstrate a direct association between EGFR-TKD and Rheb that is independent of EGFR’s tyrosine kinase activity.

### EGFR is a GEF for Rheb

**Fig. 3:**
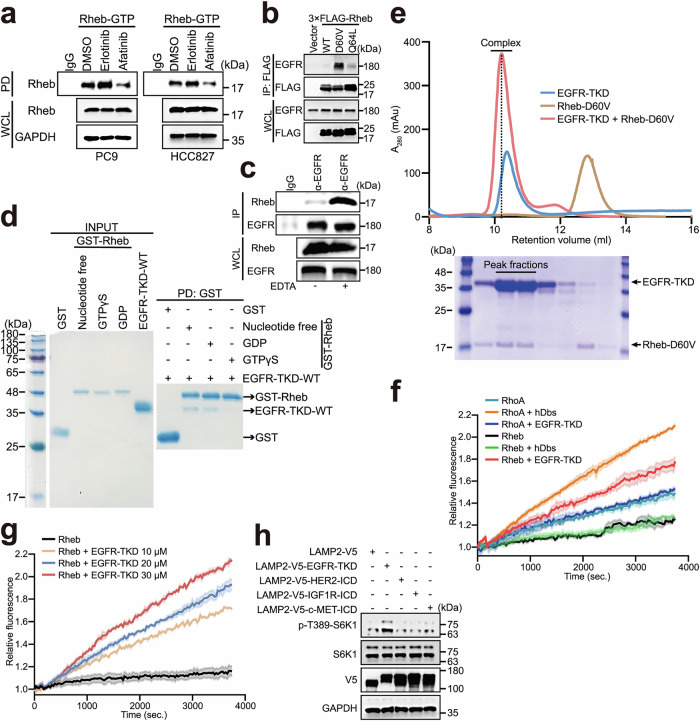
EGFR is a GEF for Rheb. **a** Afatinib decreases the level of GTP-bound Rheb in PC9 and HCC827 cells. PC9 and HCC827 cells treated with or without 25 nM erlotinib or 25 nM afatinib for 12 h, were subjected to immunoprecipitation using Rheb-GTP agarose and analyzed by western blotting. **b** The Rheb-D60V mutant preferentially interacts with endogenous WT EGFR. HEK-293T cells were transfected with empty vector, 3× FLAG-Rheb-WT, -D60V, or -Q64L as indicated, subjected to immunoprecipitation, and analyzed by western blotting. **c** EDTA increases the interaction between EGFR and Rheb at endogenous levels. HeLa cells were lysed in the absence or presence of EDTA, subjected to immunoprecipitation, and analyzed by western blotting. **d** EGFR preferentially interacts with nucleotide-free or GDP-bound Rheb in vitro. Purified EGFR-TKD was incubated with GST, nucleotide-free GST-Rheb, GST-Rheb with GDP, or GST-Rheb with GTP as indicated, and then precipitated with GST beads and subjected to SDS-PAGE analysis. Coomassie blue staining is shown. **e** Top, the reconstitution peaks in the size-exclusion chromatography analysis of the purified EGFR-TKD-WT and Rheb-D60V complex. The reconstitution peak for the complex is shown by a dotted line and observed at 10.22 mL. Bottom, SDS-PAGE analysis of each reconstitution sample. The peak for the complex of EGFR-TKD and Rheb is indicated. **f** EGFR induces Rheb nucleotide exchange from the GDP-bound to the GTP-bound state. A guanine nucleotide exchange assay was performed in vitro using purified EGFR-TKD and Rheb. The relative fluorescence reflects the guanine nucleotide exchange activity. The initial fluorescence intensity was set to 1. Human RhoGEF Dbs (hDbs) and RhoA were used as positive controls. Curves are representative of three independent experiments. Data are presented as means ± SEM. **g** EGFR induces Rheb nucleotide exchange in a dose-dependent manner. An in vitro guanine nucleotide exchange assay was performed using purified Rheb and a concentration gradient of EGFR-TKD-WT as indicated. **h** LAMP2-V5-HER2-ICD, LAMP2-V5-IGF1R-ICD, or LAMP2-V5-c-MET-ICD failed to activate mTORC1. HEK-293T cells stably expressing LAMP2-V5-EGFR-TKD-WT, LAMP2-V5-HER2-ICD, LAMP2-V5-IGF1R-ICD, or LAMP2-V5-c-MET-ICD were serum-starved for 24 h and analyzed by western blotting.

Considering the dependency of mTORC1 activation on the nucleotide-binding status of Rheb,^13,51^ we hypothesized that the aforementioned differential efficacies of EGFR-TKIs may be a result of their influence on GTP-bound Rheb. To test this idea, we performed Rheb-GTP pull-down assays.^52–54^ We found a marked decrease in Rheb-GTP levels in cells treated with afatinib compared to those treated with erlotinib (Fig. 3a).

As a central activator of mTORC1, Rheb functions as a molecular switch that cycles between the GTP-bound and GDP-bound forms.^55^ We were curious about whether EGFR itself affects the nucleotide-loading states of Rheb. Two Rheb mutants, Rheb-D60V mimicking the GDP-bound inactive form and Rheb-Q64L mimicking the GTP-bound constitutively active form, were used (Supplementary information, Fig. S6a, b). Strikingly, EGFR immunoprecipitated efficiently with Rheb-D60V, but only weakly with Rheb-Q64L (Fig. 3b). Accordingly, the association between EGFR and Rheb was strongly enhanced in cell lysates supplied with ethylenediaminetetraacetic acid (EDTA; Fig. 3c), a magnesium ion chelator disfavoring nucleotide loading.^56^ Moreover, purified EGFR-TKD preferentially interacted with nucleotide-free and GDP-bound Rheb over GTP-bound Rheb in vitro (Fig. 3d; Supplementary information, Fig. S6c). Further analysis using size-exclusion chromatography confirmed that purified EGFR-TKD and Rheb-D60V formed a stable complex in solution (Fig. 3e). These results prompted us to investigate the possibility of EGFR-TKD being a Rheb-GEF. In an in vitro nucleotide exchange assay, EGFR-TKD-WT triggered the exchange of a fluorescent GTP analog, *N*-methylanthraniloyl-GTP (*N*-MAR-GTP), for GDP-bound Rheb with efficiency comparable to that of RhoA and its GEF Dbs (Fig. 3f; Supplementary information, Table S2), in an EGFR-TKD concentration-dependent manner (Fig. 3g; Supplementary information, Table S2). Taken together, these results suggest that EGFR is a GEF for Rheb.

To ascertain the specificity of this mechanism for EGFR, we investigated whether the intracellular domains (ICDs) of other RTKs localized on lysosomes could also activate mTORC1. We constructed fusion proteins by attaching the lysosome-associated protein LAMP2-V5 to the N-termini of EGFR-TKD, HER2-ICD (amino acids 676–1255; an ErbB family member similar to EGFR), IGF1R-ICD (amino acids 960–1367; a well-known RTK involved in mTORC1 activation), and c-MET-ICD (amino acids 956–1390). As illustrated in Fig. 3h, LAMP2-V5-HER2-ICD, LAMP2-V5-IGF1R-ICD, and LAMP2-V5-c-MET-ICD did not activate mTORC1 despite their localization on lysosomes (Supplementary information, Fig. S7a). Therefore, lysosome-localized EGFR-TKD functions as a specific Rheb-GEF that directly activates mTORC1.

### EGFR-Glu804 is a potential glutamic finger for GEF activity

**Fig. 4:**
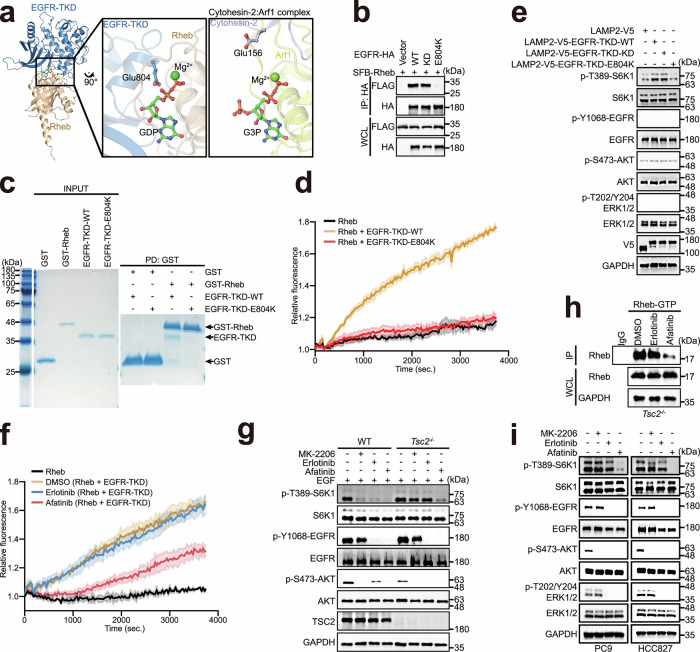
EGFR-Glu804 is a potential glutamic finger indispensable for GEF activity. **a** AlphaFold2-Multimer prediction of EGFR-TKD complexed with Rheb (amino acids 1–184). Glu804 of EGFR is shown as a stick-and-ball model and is in close proximity to the nucleotide-binding pocket of Rheb (left), which is similar to the cytohesin-2–Arf1 complex structure (PDB: 1R8Q). GDP Guanosine diphosphate, G3P Guanosine-3’-monophosphate-5’-diphosphate. **b** EGFR-E804K does not bind Rheb in cells. HEK-293T cells stably expressing SFB-Rheb were transfected with HA-tagged EGFR-WT, -KD, or -E804K, then subjected to immunoprecipitation using anti-HA antibody, and analyzed by western blotting. **c** EGFR-E804K does not directly bind Rheb in vitro. Purified EGFR-TKD-WT or -E804K was incubated with GST or GST-tagged Rheb as indicated, precipitated with GST beads, and subjected to SDS-PAGE. Coomassie blue staining is shown. **d** EGFR-E804K failed to induce Rheb nucleotide exchange. An in vitro guanine nucleotide exchange assay was performed using purified EGFR-TKD-WT or -E804K and Rheb as indicated and analyzed as described in Fig. 3f. **e** LAMP2-V5-EGFR-TKD-WT or -KD, but not LAMP2-V5-EGFR-TKD-E804K, triggers the activation of mTORC1. HEK-293T cells stably expressing the indicated plasmids were serum-starved for 24 h and analyzed by western blotting. **f** Afatinib, but not erlotinib, impairs the EGFR-mediated Rheb nucleotide exchange. An in vitro guanine nucleotide exchange assay was performed using purified Rheb and EGFR-TKD-WT with the addition of erlotinib or afatinib as indicated and analyzed as described in Fig. 3f. **g** Afatinib inhibits mTORC1 activation in TSC2-deficient MEFs. WT and TSC2-deficient MEFs were serum-starved, treated with MK-2206 (10 μM), erlotinib (10 μM), or afatinib (10 μM) for 24 h, stimulated with 100 ng/mL EGF for 30 min and analyzed by western blotting. **h** Afatinib decreases the level of GTP-bound Rheb. TSC2-deficient MEFs treated with or without 10 μM erlotinib or 10 μM afatinib as indicated for 24 h were subjected to immunoprecipitation using Rheb-GTP agarose and analyzed by western blotting. **i** MK-2206 does not inhibit mTORC1 activation in PC9 and HCC827 cells. PC9 and HCC827 cells were treated with MK-2206 (1 μM), erlotinib (25 nM), or afatinib (25 nM) for 12 h as indicated, and analyzed by western blotting.

To understand how EGFR mediates the nucleotide exchange of Rheb, we modeled the structure of the EGFR-TKD and Rheb complex using AlphaFold2.^57^ The resulting model indicated that EGFR-TKD may interact with Rheb at its nucleotide-binding pocket (Fig. 4a; Supplementary information, Fig. S8). The side chain of Glu804 on EGFR protrudes towards the magnesium ion and nucleotide, resembling the mechanism by which Arf1-GEF cytohesin-2 mediates nucleotide exchange on Arf1 via a glutamic finger (Fig. 4a).^58,59^ When Glu804 was mutated to lysine, the association between EGFR and Rheb was significantly weakened compared to the WT or KD counterparts in cells (Fig. 4b). Furthermore, the direct binding of EGFR-TKD to Rheb was abolished in vitro (Fig. 4c), indicating that Glu804 in EGFR is a key residue for the EGFR–Rheb interaction. Consistently, EGFR-TKD-E804K failed to catalyze the nucleotide exchange of Rheb in vitro (Fig. 4d; Supplementary information, Table S2). In addition, cells expressing LAMP2-V5-EGFR-TKD-WT and -KD, but not LAMP2-V5-EGFR-TKD-E804K, effectively triggered mTORC1 without activating EGFR and its downstream signals, such as AKT and ERK1/2 (Fig. 4e; Supplementary information, Fig. S7b).

Previous structural studies have elucidated distinct binding patterns of erlotinib (PDB: 4HJO)^60^ and afatinib (PDB: 4G5J)^61^ to EGFR, which may differentially modulate the EGFR–Rheb interaction. Indeed, afatinib, but not erlotinib, could impair the EGFR-catalyzed nucleotide exchange of Rheb in vitro (Fig. 4f; Supplementary information, Table S2), consistent with our results showing disrupted EGFR–Rheb interaction (Fig. 2b, d) and a reduced level of GTP-bound Rheb (Fig. 3a) in the presence of afatinib. Considering that EGFR is encoded by *ErbB1*, which was initially identified in vertebrates,^62^ we analyzed multiple sequence alignments and found that Glu804 in human EGFR is strictly conserved in vertebrates (Supplementary information, Fig. S9).

As a key regulator in the PI3K-AKT-TSC2-Rheb-mTORC1 pathway, genetic loss of TSC2, the GAP of Rheb, leads to constitutive activation of mTORC1, which is independent of PI3K kinases.^63,64^ We examined mTORC1 activation in TSC2-deficient MEFs treated with AKT inhibitor MK-2206 or EGFR-TKIs. Although all compounds reduced EGF-induced mTORC1 activation in WT MEFs, only afatinib remained effective in TSC2-deficient MEFs (Fig. 4g). The level of GTP-bound Rheb dramatically decreased in afatinib-treated TSC2-deficient MEFs, but not in erlotinib-treated cells (Fig. 4h). In addition, for EGFR mutant cells, treatment with MK-2206 or erlotinib showed limited influence on mTORC1 activation (Fig. 4i). These results indicate that Glu804 is a potential glutamic finger in EGFR’s function as a Rheb-GEF.

### Aberrant Rheb-GEF activity of EGFR impairs mTORC1 activation

**Fig. 5:**
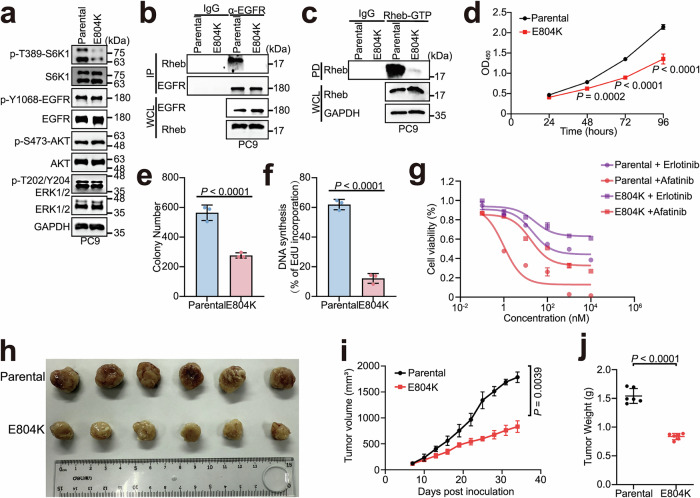
Aberrant Rheb-GEF activity of EGFR impairs mTORC1 activation and cell growth. **a** mTORC1 activation was inhibited in EGFR-E804K knock-in PC9 cells. EGFR-E804K knock-in PC9 cells were analyzed by western blotting. **b** The EGFR–Rheb interaction was disrupted in EGFR-E804K knock-in cells. EGFR-E804K knock-in PC9 cell lysates were subjected to immunoprecipitation using IgG or anti-EGFR antibody and analyzed by western blotting. **c** The level of GTP-bound Rheb was decreased in EGFR-E804K knock-in cells. EGFR-E804K knock-in PC9 cell lysates were subjected to immunoprecipitation using Rheb-GTP agarose and analyzed by western blotting. **d** Cell vitality was decreased in EGFR-E804K knock-in cells. CCK-8 assays were performed to examine the proliferation in parental and EGFR-E804K knock-in PC9 cells (*n* = 4). Two-way ANOVA was used. **e**, **f** Cell proliferation was diminished in EGFR-E804K knock-in cells. Colony formation (**e**) and EdU (**f**) assays were used to evaluate the proliferation ability of parental and EGFR-E804K knock-in PC9 cells (*n* = 3). Two-tailed unpaired *t*-test. **g** EGFR-E804K knock-in PC9 cells exhibit reduced sensitivity to afatinib compared to parental cells. Parental and EGFR-E804K knock-in PC9 cells were treated with indicated concentrations of erlotinib or afatinib for 72 h (*n* = 3). The IC_50_ values were as follows: 20.59 nM and 0.97 nM for parental cells treated with erlotinib and afatinib, respectively; 25.89 nM and 17.44 nM for EGFR-E804K knock-in cells treated with erlotinib and afatinib, respectively. **h** Representative parental and EGFR-E804K knock-in PC9 tumors surgically removed. **i** EGFR-E804K knock-in effectively inhibited tumor growth in vivo. Tumor volumes in mice were measured and calculated at specified time intervals following implantation. Two-tailed unpaired *t*-test. **j** EGFR-E804K knock-in tumors weighed less than those derived from parental cells. Five weeks after implantation, the mice were euthanized, and the tumors were excised and weighed. Two-tailed unpaired *t*-test was used.

To elucidate the impact of EGFR’s Rheb-GEF function on mTORC1 activation in the context of EGFR mutation, we introduced an E804K mutation to EGFR in PC9 cells and generated a homozygous EGFR-E804K knock-in model (Supplementary information, Fig. S10a). The subcellular distribution of EGFR with LAMP-1 was not affected by E804K mutation, even under treatment of erlotinib or afatinib. (Supplementary information, Fig. S10b). Although the phosphorylation status of EGFR and downstream signaling mediators (AKT and ERK1/2) also remained intact, a notable reduction in mTORC1 activation was observed (Fig. 5a), pinpointing that the E804K mutation specifically affects the Rheb-GEF activity without impairing the intrinsic tyrosine kinase activity of EGFR. In addition, the interaction between EGFR and Rheb in the EGFR-E804K knock-in cells was disrupted, which resulted in a remarkable decrease in the amount of GTP-bound Rheb compared with that in the parental cells (Fig. 5b, c).

To substantiate these findings, we extended our examination to cells harboring WT EGFR and generated EGFR-E804K knock-in HEK-293T cells (Supplementary information, Fig. S10c). Upon EGF stimulation, mTORC1 activation was greatly compromised in E804K knock-in cells (Supplementary information, Fig. S10d). This diminished mTORC1 activation coincided with a decreased association between EGFR and Rheb, as well as a reduced level of GTP-bound Rheb compared with those in WT EGFR cells (Supplementary information, Fig. S10e, f).

Inhibition of mTORC1 restricts cell growth. We examined the cell proliferation of EGFR-E804K knock-in PC9 cells. As shown by CCK-8 and colony formation assays, the proliferation of EGFR-E804K knock-in cells was strongly hampered (Fig. 5d, e), which was further corroborated by the results of EdU staining (Fig. 5f; Supplementary information, Fig. S10g). Notably, compared to parental cells, EGFR-E804K knock-in cells exhibited dramatically decreased sensitivity to afatinib (IC_50_ of 17.44 nM vs 0.97 nM), while their sensitivity to erlotinib was only slightly reduced (IC_50_ of 20.59 nM vs 25.89 nM) (Fig. 5g). Additionally, in xenograft models established by subcutaneous implantation of either PC9-parental or EGFR-E804K knock-in cells, the tumor growth and weight of EGFR-E804K knock-in xenografts were significantly reduced (Fig. 5h–j). These results suggest that Rheb-GEF activity of EGFR is crucial for mTORC1 activation and cell growth.

### Discovery of BIEGi-1, a dual inhibitor of the GEF and kinase activities of EGFR, efficiently suppresses cancer cell growth

**Fig. 6:**
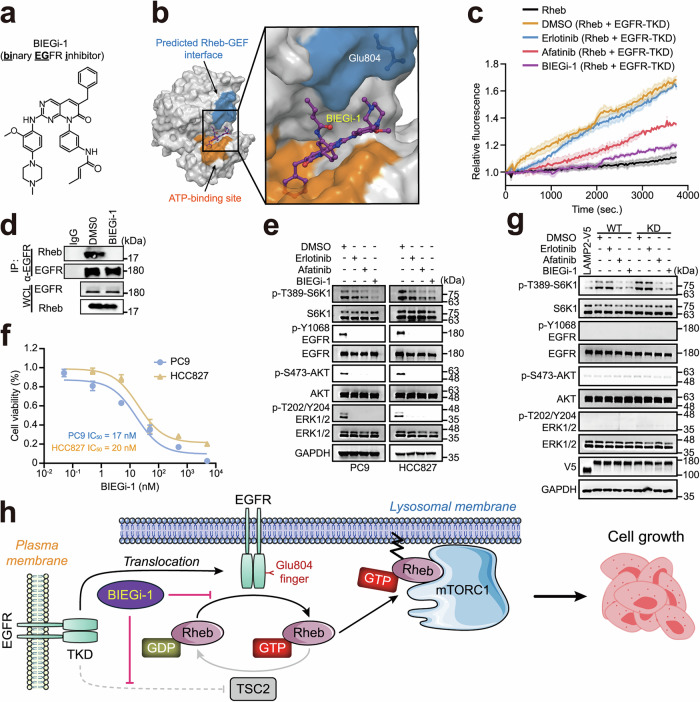
BIEGi-1 suppresses cancer cell growth. **a** Chemical structure of BIEGi-1. **b** Structural model of EGFR-TKD complexed with BIEGi-1. EGFR-TKD is shown in surface representation and BIEGi-1 in ball-and-stick. The ATP-binding site (orange) and predicted Rheb-GEF interface (blue) of EGFR are indicated. **c** BIEGi-1 impairs the EGFR-mediated Rheb nucleotide exchange. An in vitro guanine nucleotide exchange assay was performed using purified Rheb and EGFR-TKD-WT with the addition of erlotinib, afatinib or BIEGi-1 and analyzed as described in Fig. 3f. **d** BIEGi-1 abolishes the interaction between EGFR and Rheb in cells. PC9 cells treated with or without 25 nM BIEGi-1 for 12 h were subjected to immunoprecipitation and analyzed by western blotting. **e** BIEGi-1 inhibits mTORC1 activation in EGFR-mutated cells. PC9 and HCC827 cells were treated with 25 nM erlotinib, afatinib, or BIEGi-1 for 12 h as indicated, and analyzed by western blotting. **f** CCK8 assays of PC-9 and HCC827 cells exposed to BIEGi-1. The IC_50_ values were 17 nM and 20 nM for PC9 and HCC827 cells, respectively. Data points represent the means ± SD (*n* = 3). **g** BIEGi-1 and afatinib, but not erlotinib, inhibit the activation of mTORC1 induced by LAMP2-V5-EGFR-TKD. HEK-293T cells stably expressing the indicated plasmids were serum-starved, treated with 200 nM BIEGi-1, erlotinib or afatinib for 24 h, and analyzed by western blotting. WT and KD refer to LAMP2-V5-EGFR-TKD-WT and LAMP2-V5-EGFR-TKD-KD, respectively. **h** Model illustrating that lysosomal EGFR acts as a GEF for Rheb, with Glu804 serving as a glutamic finger to activate mTORC1. Inhibition of both GEF and kinase activities of EGFR by BIEGi-1 restricts mTORC1 activity, leading to impaired cell growth.

Considering the unignorable clinical side effects of afatinib, we tried to design ab initio new types of EGFR-targeting small-molecule compounds with binary efficacy in inhibiting both kinase and Rheb-GEF activities. After multiple rounds of optimization and screening, we identified a highly potent and selective compound, binary EGFR inhibitor (BIEGi-1), which has a backbone that is distinct from afatinib (Fig. 6a). According to molecular docking results, the potential binding site of BIEGi-1 on EGFR overlaps with the EGFR–Rheb interface (Fig. 6b). Notably, BIEGi-1 exhibited stronger inhibition on EGFR-catalyzed nucleotide exchange than afatinib in vitro (Fig. 6c; Supplementary information, Table S2) and effectively disrupted the interaction between EGFR and Rheb in cells (Fig. 6d). In EGFR-mutant cells, treatment with BIEGi-1 resulted in robust inhibition of the kinase activity of EGFR and mTORC1 activation (Fig. 6e). BIEGi-1 also exhibited strong anti-proliferative effects on PC9 and HCC827 cells, with IC_50_ values of 17 nM and 20 nM, respectively (Fig. 6f). Furthermore, BIEGi-1 prevented lysosomal EGFR-TKD-WT or -KD from activating mTORC1 in a similar manner as afatinib did (Fig. 6g). Together, these data highlight the value of simultaneously targeting EGFR’s GEF and kinase activities to restrict the growth of cancer cells harboring EGFR mutations.

## Discussion

In this study, we delineated a hitherto uncharacterized mechanism by which EGFR triggers mTORC1 in a *cis* manner. This mechanism is distinct from the canonical EGFR-PI3K-AKT pathway that mediates dissociation of the TSC2 complex from GDP-bound Rheb. Compared to the canonical phosphorylation cascade-based pathways, this newly identified EGFR-driven mechanism possesses at least two unique features. First, the new mechanism is characterized by the physical association between EGFR and Rheb, in which EGFR directly activates Rheb by acting as a Rheb-GEF. Second, the new mechanism is independent of the kinase activity but relies on the lysosomal translocation of EGFR. Our findings, including the new role of EGFR as a new GEF for Rheb, greatly augment the fundamental knowledge of cell growth in physiological and pathological conditions.

Target of rapamycin (TOR) controls cell growth and metabolism in response to nutrients, growth factors, and cellular energy status.^65^ Nutrients, especially amino acids, are thought to be ancestral TORC1-activating inputs, as they are sufficient to activate TORC1 in unicellular organisms such as yeast.^66^ However, in multicellular organisms, TORC1 activation requires additional input from growth factors. Mechanistically, amino acids induce the translocation of mTORC1 from the cytosol to lysosomes through Rag GTPases, whereas growth factors promote the activation of the lysosomal GTPase Rheb by preventing its conversion to the GDP-bound form, eventually triggering the kinase activity of mTORC1.^67^ Thus, Rags and Rheb define the two independent arms that cooperatively regulate the mTORC1 pathway.^3^ In the past two decades, many breakthroughs have been achieved in determining the mechanisms by which amino acids activate Rags through a series of delicate sensor networks.^2,3^ On the other hand, how growth factors activate Rheb is yet incompletely understood, with the exception of the PI3K-AKT-TSC2 pathway. TSC2 inhibits mTORC1 with dual roles as both a GAP and a potential guanine nucleotide dissociation inhibitor for Rheb; it promotes GTP hydrolysis of Rheb, and then binds the resulting inactive GDP-bound Rheb to sequester it from triggering mTORC1.^16,17,68^ Upon EGF binding, activated EGFR triggers the downstream PI3K-AKT signaling to phosphorylate TSC2 and release it from Rheb, which allows the GTP reloading of Rheb essential for mTORC1 activation. Though endorsed by the fact that loss of TSC2 leads to hyperactivation of mTORC1, this mechanism apparently remains imperfect due to the absence of a key factor that resurrects inactive GDP-bound Rheb, that is to say, a Rheb-GEF. We found that, besides activating the PI3K-AKT-TSC2 pathway, EGFR also serves as a Rheb-GEF to directly trigger mTORC1. The crucial role of EGFR’s Rheb-GEF function in mTORC1 activation is substantiated by the observation that blocking the interaction between EGFR and Rheb significantly abolishes mTORC1 activation (Fig. 5a–c; Supplementary information, Fig. S10d–f). Thus, our findings uncover a pivotal step of mTORC1 activation, and complete the mechanistic scenario of growth factor-induced cell growth. In addition, ATP6AP1 has been reported recently to be another GEF for Rheb that triggers mTORC1 upon insulin stimulation,^69^ which represents another path for growth factor-induced mTORC1 activation.

A special feature of this unexpected Rheb-GEF function is that it relies on the subcellular localization of EGFR, but not on the kinase activity. Remarkably, TKDs of both WT and KD EGFR tethered to the lysosomal surface were able to trigger mTORC1 (Fig. 4e). This discovery is particularly important because it explains the observed irrelevance of tumor growth to the kinase activity of EGFR in cancer cells.^29,70^ Our data also shed light on how varied intracellular trafficking of mutant EGFR leads to constitutive activation of mTORC1. Under physiological conditions, endocytic EGFR molecules are predominantly recycled to the plasma membrane, resulting in relatively weak mTORC1 activation (Supplementary information, Fig. S3a).^50^ In contrast, cells harboring mutant EGFR exhibit aberrant endocytic trafficking, leading to predominant intracellular localization in compartments, such as the late endosome or lysosome.^40^ This causes sustained signaling and a distinct pattern of mTORC1 activation, which leads to oncogenic processes such as uncontrolled cell proliferation.

The development of inhibitors targeting mTOR or EGFR has notably advanced cancer therapy. Despite their clinical benefits, mTOR inhibitors, including rapamycin and its analogs (e.g., everolimus and temsirolimus), encounter challenges such as drug resistance, adverse effects, and limitations of efficacy.^71,72^ A known disadvantage of mTOR inhibitors is their tendency to activate AKT pathways via a feedback mechanism, which may favor tumor survival and undermine therapeutic outcomes.^73^ Similarly, EGFR inhibitors always end up with drug resistance.^74^ To overcome these barriers, combined administration of mTORC1 and EGFR inhibitors was tested in clinical trials. However, these trials were unsuccessful because of overlapping toxicities.^75^

Afatinib, as we found, exhibits a dual mechanism of action: it not only inhibits EGFR kinase activity but may also partially block the GEF activity of EGFR (Figs. 1c, 4f, g, i). While showing superior therapeutic efficacy for lung adenocarcinoma over erlotinib, the administration of afatinib at maximum tolerated doses is associated with a higher incidence of adverse events.^76,77^ Thus, the development of other binary EGFR inhibitors with low toxicity is of great clinical value. Compared to afatinib, our binary EGFR inhibitor BIEGi-1 is more effective in blocking EGFR’s Rheb-GEF activity. Given its unique chemical structure and low IC_50_ values, BIEGi-1 is a promising prototype for a new class of EGFR inhibitors. We are currently focusing on evaluating the in vivo efficacy of BIEGi-1 and its derivatives in animal models, with an eventual goal of clinical trials for cancer patients with EGFR mutations.

In summary, we demonstrate that lysosomal EGFR acts as a Rheb-GEF independent of its kinase activity to activate mTORC1, and propose that specifically targeting both kinase and Rheb-GEF activities of EGFR (e.g., BIEGi-1) is a promising therapeutic strategy for cancer patients with EGFR mutations, as illustrated in Fig. 6h.

## Materials and methods

### Antibodies and chemicals

Reagents were obtained from the following sources: antibodies against phospho-Y1068 EGFR (2234S), EGFR (4267 L), TSC2 (4308S), Rheb (13879S), mTOR (2983S), phospho-T389 S6K1 (9234S), S6K1 (9202S), phospho-S473 AKT (4060S), phospho-Thr202/Tyr204-p44/42 MAPK (Erk1/2) (4370S), 4E-BP1 (9644S), Phospho-4E-BP1 (Thr37/46) (2855S), β-actin (4970S), GFP (2956S), FLAG (14793S), HA (3724S and 2367S), V5 (13202S), and LAMP1 (9091S) from Cell Signaling Technology; an antibody against EGFR (AF231-SP) from Bio-Techne includes R&D Systems; an antibody against GAPDH (FD0063) from Fudebio-tech; an antibody against EGFR (GTX628887) from GeneTex; an antibody against AKT (10176-2-AP) and normal rabbit IgG (30000-0-AP) from Proteintech; an antibody against Rheb (H00006009-M01) from Abnova; antibodies against ERK1/2 (AF1051) and Alexa Fluor-647 (A0468) from Beyotime; antibodies conjugated with Alexa Fluor-594 (A-11005 and A-11012), Alexa Fluor-488 (A-21202 and A-21206), Alexa Fluor-647 (A32849) and Hoechst 33342 (62249) from Invitrogen; horseradish-peroxidase (HRP)-conjugated (W401B and W402B) antibodies from Promega; anti-FLAG affinity gel (B23012) from Bimake; anti-V5 agarose affinity gel antibody produced in mouse (A7345), monoclonal anti-HA-agarose antibody produced in mouse (A2095), protein A agarose (P3476), GTPγS (G8634), and GDP (G7127) from Sigma‒Aldrich; animal-free recombinant human EGF (96-AF-100-15) from PeproTech; Dyngo-4a (S7163), erlotinib HCl (S1023), afatinib (S1011), and MK-2206 2HCl (S1078) from Selleck; LY3000328 (HY-15533) from MCE; RhoGEF Exchange Assay Kit (BK100) from Cytoskeleton; an active Rheb-GTP kit (26910) from NewEastBio; antifade mounting medium (P0128M) from Beyotime.

### Cell lines and culture conditions

PC9, NCI-H1975, HEK-293T, and HeLa cells were obtained from the American Type Culture Collection. HCC827 cells were obtained from iCell. WT MEFs and TSC2-deficient MEFs were kindly provided by Dr. Hongbing Zhang (Chinese Academy of Medical Sciences). HCC827 cells were cultured in RPMI-1640 (Gibco) with 10% fetal bovine serum (FBS) supplemented with 100 U/mL penicillin and streptomycin. Other cells were cultured in DMEM (Gibco) with 10% FBS supplemented with 100 U/mL penicillin and streptomycin. All cell lines were maintained at 37 °C and 5% CO_2_. The cells were free of mycoplasma contamination.

### Lentivirus production and infection

Lentiviruses were made by co-transfecting pSin-EF2-cDNA with the psPAX2 (gag, pol) and pMD2G packaging plasmids into HEK-293T cells using transfection reagent PEI. The viral supernatant was collected after transfection for 48 h and filtered using a 0.45-μm syringe filter.

For lentivirus infection, target cells were seeded with the virus and 10 μg/mL polybrene. After 24 h, the virus was removed, and the cells were supplemented with fresh medium containing puromycin for selection. Experiments were performed after infection for 72 h.

### DNA transfection

For DNA construct transfection, all experiments were carried out using Lipofectamine™ 3000 (Invitrogen, L3000015) according to the manufacturer’s instructions.

### Cell treatment

For serum starvation, cells were seeded into appropriate plates 24 h prior to experiments, rinsed twice with PBS, and incubated in DMEM without FBS for the indicated time.

For stimulation with EGF, serum-starved cells were stimulated with DMEM containing the indicated concentration of EGF at the indicated time.

Erlotinib, afatinib, MK2206, BIEGi-1, Dyngo-4a, and LY3000328 were dissolved in sulfoxide (DMSO) and added into DMEM to the final concentration.

### Compound

BIEGi-1, described in Patent Publication WO/2014/079232 (International Application No. PCT/CN2013/080758), was designed and synthesized by Ke Ding’s group.

### Generation of EGFR-E804K knock-in PC9 and HEK-293T cells

The knock-in tests were carried out as described previously.^78^ EGFR-targeting sgRNAs were created, and the efficient sgRNA was selected and co-transfected with a designed DNA donor containing an E804K mutation into PC9 or HEK-293T cells. The EGFR donor template was designed for integration into the pSIN vector without the EF1α promoter. It included the sequence encoding EGFP, as well as an internal ribosome entry site upstream to boost the effectiveness of positive clone selection. Three days after transfection, the MoFlo Astrios EQ Cell Sorter (Beckman) was used to sort Alexa Fluor-488-positive single cells into 96-well plates. The single clones were then confirmed by PCR and Sanger sequencing. The sequences of the donor template and sgRNA for E804K knock-in are reported in Supplementary information, Table S3.

### GTP-binding assays

GTP-binding assays were performed as described previously.^79^ Briefly, HEK-293T cells stably expressing Rheb-WT, Rheb-D60V, or Rheb-Q64L were harvested at ~90% confluence. Cells were suspended in binding buffer (20 mM HEPES (pH 8), 150 mM NaCl, 10 mM MgCl_2_) containing a protease inhibitor cocktail and lysed using three freeze‒thaw cycles. The lysates were centrifuged at 14,000× *g* at 4 °C for 10 min, and the supernatants were incubated with 100 μL of a GTP-agarose suspension (Sigma Aldrich, G9768) for 1 h with rotation at 4 °C. The beads were washed three times with binding buffer and suspended in 40 μL of sample buffer. The proteins were boiled and subjected to SDS-PAGE and western blotting.

### Rheb pull-down activation assay

The Rheb-GTP level was measured using the Rheb Activation Assay Kit according to the manufacturer’s instructions. Briefly, cells were washed twice with ice-cold PBS. Ice-cold 1× assay/lysis buffer (50 mM Tris-HCl (pH 8), 150 mM NaCl, 10 mM MgCl_2_, 1 mM EDTA, 1% Triton X-100) containing a protease inhibitor cocktail was added for 20 min. The lysates were cleared by centrifugation at 12,000× *g* at 4 °C for 10 min. Next, 1 μL of anti-Rheb-GTP antibody was added to the cell lysates and incubated with 20 μL of a resuspended bead slurry at 4 °C for 1 h with gentle agitation. The beads were washed three times with 0.5 mL of 1× assay/lysis buffer and suspended in sample buffer. The proteins were denatured and subjected to SDS-PAGE and western blotting.

### Western blotting and immunoprecipitation

For western blotting, cells were rinsed once with ice-cold PBS and immediately lysed in RIPA lysis buffer (50 mM Tris-HCl (pH 7.5), 150 mM NaCl, 1 mM EDTA, 1% NP40) containing Protease Inhibitor Cocktail set І (Calbiochem, 539131) and Phosphatase Inhibitor Cocktail set II (Calbiochem, 524625). Cell lysates were cleared by centrifugation at 12,000 rpm for 20 min at 4 °C. Protein levels in the cell lysate were measured by the Bradford assay.

For FLAG, V5, or HA immunoprecipitation, cells were rinsed once with ice-cold PBS and lysed with HEPES lysis buffer (40 mM HEPES (pH 7.4), 2.5 mM MgCl_2_) containing protease inhibitor. The soluble fraction of the cell lysates was isolated by centrifugation at 12,000 rpm for 10 min. The FLAG-M2, V5, or HA affinity gel was equilibrated with HEPES lysis buffer. A total of 20 μL of the indicated beads was added to the cleared lysates and incubated with rotation overnight at 4 °C.

For endogenous immunoprecipitation, cells were rinsed once with ice-cold PBS and lysed with HEPES lysis buffer. The HEPES lysis buffer was supplemented with 12.5 mM EDTA as described when needed. The cell lysates were cleared by centrifugation at 12,000 rpm at 4 °C for 10 min. Protein A agarose beads were washed three times with HEPES lysis buffer, and then anti-EGFR antibody or control rabbit IgG was added to the cell lysates with 50 μL of a 50% slurry of beads, followed by incubation overnight at 4 °C. Immunoprecipitates were washed five times with HEPES lysis buffer containing 150 mM NaCl.

The cell lysates and immunoprecipitated proteins were boiled in gel loading buffer for 10 min and resolved by 10%‒15% SDS-PAGE depending on the molecular mass of the target protein. The gels were transferred to Immobilon-P PVDF membranes (Millipore), which were then blocked in PBS with 5% nonfat milk and 0.1% Tween-20 and probed with primary antibodies overnight at 4 °C. After incubation with HRP-conjugated secondary antibodies for 1 h, Clarity ECL substrate (Bio-Rad) was used for detection with a MiniChemi chemiluminescence imager (SAGECREATION, Beijing).

For Lyso-IP, we utilized PC9 cells stably expressing HA-tagged TMEM192 to isolate lysosomes as previously described.^80^ Briefly, cells were washed with PBS, scraped, and collected by centrifugation. The cell pellet was then resuspended in KPBS buffer (136 mM KCl, 10 mM KH_2_PO_4_, Protease Inhibitor Cocktail Set I, and Phosphatase Inhibitor Cocktail Set II, pH 7.25) and homogenized. The homogenate was centrifuged at 1000× *g* for 3 min at 4 °C to separate the supernatant. The supernatant was subsequently incubated with 20 μL of Anti-HA Magnetic Beads (Beyotime) for 1 h with rotation. After incubation, the beads were washed six times with KPBS. Lysosomes bound to the beads were eluted using HA-peptide, and the eluent was transferred to a polylysine-coated fluorescent dish for further fluorescence staining analysis.

### Immunofluorescence

Cells were seeded on glass-bottom culture dishes (NEST Biotechnology, 801002) for 24 h prior to the experiments. Cells were treated as indicated, rinsed twice with PBS, and fixed using 4% paraformaldehyde (PFA) for 10 min at room temperature. The cells were then rinsed three times with PBS and permeabilized with 0.5% Triton X-100 in PBS for 10 min. After being rinsed three times with PBS, the cells were blocked with goat serum for 30 min at room temperature and incubated with primary antibodies overnight at 4 °C. The dishes were rinsed three times with PBS and then labeled with fluorescently conjugated secondary antibodies for 2 h at room temperature in the dark. Nuclei were stained with Hoechst 33342 for 1 min (Molecular Probes, Invitrogen). The dishes were rinsed with PBS three times and mounted using antifade mounting medium. All confocal microscopy was performed using a laser scanning system (ZEISS, LSM880, ZEN2.6, 63× oil lens, or Nikon, CSU-W1, 60× oil lens).

Protein co-localization in confocal microscopy experiments was quantified using the ImageJ software co-localization tool. For each experimental condition, 3–5 separate, representative confocal images were analyzed. Pearson’s correlation coefficient was calculated for individual cells to assess co-localization. The results are presented as means ± standard deviation (SD). Statistical significance was determined using either Student’s *t*-test or one-way ANOVA, performed in Prism 9.5.0.

### SIM

Super-resolution SIM images were taken using a Nikon N-SIM system with a 100× oil immersion objective lens, 1.49 NA (Nikon). Images were captured using Nikon NIS-Elements and were reconstructed using slice reconstruction in NIS-elements.

### Cell proliferation assays

PC9 and HCC827 cells were suspended and seeded in 96-well microplates at a concentration of 2000 cells per well for experiments with Cell Counting Kit-8 (CCK-8) (GOONIE, 100-106). Subsequently, the cells were incubated overnight. The cellular viability was evaluated by quantifying the absorbance of the converted dye at a wavelength of 450 nm. In addition, for Figs. 5g and 6f, 24 h after planting, suitable medium with or without indicated inhibitors was introduced into each well. Control cells were subjected to the identical concentration of DMSO. The absorbance was measured 72 h after treatment.

In the colony formation assay, the indicated cells were placed in 6-well plates at a density of 2000 cells per well. After incubation for more than 10 days, the cell colonies were treated with PFA to prevent further growth and then stained with crystal violet. ImageJ was utilized to compute the colonies. The experiment was conducted three times.

The BeyoClick™ EdU Cell Proliferation Kit with Alexa Fluor-488 (Beyotime, C0071) was utilized for EdU staining according to the directions provided by the manufacturer. The specified cells were placed in 24-well plates and kept for 24 h prior to the experiment. Each well was treated with 10 μM of EdU reagent and incubated for 2 h to mark the cells. Following three rounds of treatment with PBS, the cells were immobilized in a 4% PFA solution for 15 min. Subsequently, they were made permeable using 0.3% Triton X-100 for an additional 15 min. Finally, the cells were exposed to the click-reaction reagent for 30 min at room temperature in a light-restricted environment. A single unit of Hoechst33342 reagent was utilized to counterstain the nucleus. The fluorescent microscope instrument used for observing the staining result was the Nikon CSU-W1. The data were obtained using NIS-Elements F v4.0 software.

### Xenograft tumor model

The animal study was approved by the Animal Research Committee of Sun Yat-sen University Cancer Center (L025501202410005) and was performed following the established guidelines and principles.

Female BALB/c nude mice (18–20 g, 4-week-old) were purchased from Zhuhai Bestest Biotechnology Co., Ltd. (Zhuhai, China). The mice were randomly allocated into two groups, with six mice per group. For tumor growth assays, 5 × 10^6^ PC9-parental or EGFR-E804K knock-in cells were subcutaneously injected into the armpit of the mice. Starting one week after injection, tumor volumes were measured every three days using a Vernier caliper, calculated using the formula: V (volume) = (width^2 × length × π)/6. Following 5 weeks of growth, the mice were euthanized using 100% CO_2_ at a flow rate of 30%–70% of the chamber volume per minute. Tumors were harvested, and their weights and volumes were measured with an electronic balance and Vernier caliper, respectively.

### Protein expression and purification

An N-terminal GST fusion protein consisting of human Rheb-WT or -D60V (amino acids 1–169) was expressed in the *Escherichia coli* BL21(DE3) strain using the pGEX-6P-1 vector and grown in Luria-Bertani (LB) medium with 100 μg/mL ampicillin. Protein expression was induced with 1 mM isopropyl-β-D-thiogalactopyranoside (IPTG, Sigma‒Aldrich, St. Louis, MO, USA) at OD600 0.6, and the bacteria were cultured at 16 °C overnight. The bacteria were harvested and resuspended in 50 mM HEPES (pH 7.5), 500 mM NaCl, 5 mM MgCl_2_, 1 mM dithiothreitol (DTT), and 1 mM phenylmethanesulfonylfluoride. The following protein purification steps were carried out at 4 °C. The bacterial suspension was homogenized by high-pressure cell disruption, followed by centrifugation at 20,000× *g* for 1 h. The supernatant was 0.45-µm filtered and exposed to GST-tag purification resin (Beyotime, P2253) for 2 h, before which the beads were equilibrated three times with an equilibrated buffer (50 mM HEPES (pH 7.5), 500 mM NaCl, 5 mM MgCl_2_, 1 mM DTT). GST-Rheb was eluted using wash buffer (50 mM HEPES (pH 7.5), 5 mM MgCl_2_, 1 mM DTT) supplemented with 10 mM glutathione (Sigma, G4251) and subsequently concentrated by centrifugal ultrafiltration using wash buffer. When necessary, Rheb was cleaved from GST on the beads by adding GST-bound beads with HRV3C protease for incubation overnight. Proteins were eluted the next day and further purified by size-exclusion chromatography using a Superdex 200 pg column (GE Healthcare). Peak fractions were collected and concentrated, and the concentration was determined by UV absorption at 280 nm, followed by flash freezing in liquid nitrogen for storage at –80 °C.

Human EGFR (amino acids 696–1022) was cloned into the pFastBac1 vector (Thermo Fisher Scientific) using restriction sites *Eco*RI and *Xba*I to enable the infection of Sf9 insect cells through the Bac-to-Bac baculovirus expression system according to the manufacturer’s instructions. Sf9 cells were grown in suspension in Sf-900™ II SFM medium (Gibco) to an appropriate density and infected with the recombinant baculovirus. The cells were infected for 60–72 h at 28 °C, and the expression of EGFR was identified by immunofluorescence. The cells were harvested by centrifugation at 2000× *g* for 15 min and homogenized by high-pressure cell disruption in buffer A (50 mM HEPES (pH 7.5), 500 mM NaCl, 30 mM imidazole, 5 mM MgCl_2_, 5% glycerol). The extraction mixture was centrifuged at 30,000× *g* at 4 °C for 1 h. The cleared supernatant was filtered and applied to an Ni-NTA column (GE Healthcare). Ni-NTA purification was carried out in buffer B (50 mM HEPES (pH 7.5), 5 mM MgCl_2_, 1 mM DTT) supplemented with different concentrations of imidazole (10, 30, and 300 mM) for equilibration, washing, and elution, respectively. The supernatant was subsequently loaded onto a HiLoad 16/600 Superdex 200 pg column (GE Healthcare). Peak fractions containing the protein were collected and concentrated.

For the purification of the EGFR-TKD and Rheb-D60V complex, EGFR-TKD was incubated with a 3-fold molar excess of Rheb-D60V in buffer B containing 1 mM EDTA overnight at 4 °C. The complex was purified by size exclusion chromatography in buffer B.

### Guanine nucleotide-binding assay

For guanine nucleotide binding on purified GST-Rheb or Rheb, the proteins were incubated in loading buffer (40 mM HEPES (pH 7.5), 1 mM DTT, 20 mM EDTA) with 1 mM GTPγS or GDP at 30 °C for 30 min. Subsequently, the proteins were added to 20 mM MgCl_2_ at 25 °C and incubated for 20 min. Next, the proteins were concentrated and eluted.

### High-performance liquid chromatography (HPLC) analysis

The reversed-phase Hypersil ODS-2 C18 column (250 × 4.6 mm, Thermo Fisher Scientific) can detect nucleotides by absorption at 254 nm and separate GTP and GDP based on their differences in charge capacity. In this experiment, the running buffer (10 mM tetrabutylammonium bromide, 100 mM potassium phosphate, pH 6.5, and 7.5% acetonitrile) was used as the mobile phase. GST-tagged Rheb was diluted to 50 μM by Gel buffer, denatured at 100 °C for 10 min, and centrifuged at 15,060 rpm for 10 min. The supernatant was tested by RP-HPLC at a speed of 1 mL/min for 10 min. Standard products of 100 μΜ GDP and 100 μM GTP were used for comparison.

### In vitro binding assay

For the in vitro binding assay, 1 μM recombinant purified GST or GST-Rheb was incubated with 1 μM EGFR in binding buffer (50 mM HEPES (pH 7.5), 2 mM DTT, 5 mM MgCl_2_, 1 mg/mL bovine serum albumin (BSA)) for 16 h at 4 °C with glutathione beads. To terminate the binding assays, samples were washed three times with ice-cold wash buffer (50 mM HEPES (pH 7.5), 5 mM MgCl_2_, 1 mM DTT) and assessed by Coomassie blue staining.

For the interaction between EGFR and GST-Rheb at different guanine nucleotide-bound states, 15 μg of EGFR and 15 μg of nucleotide-loaded, nucleotide-free GST-Rheb or GST were gently rotated at 4 °C overnight. The samples were prepared as indicated above.

For the inhibition assay, 100 μM erlotinib or afatinib was added to the mixture containing 1 μM GST or GST-Rheb and EGFR. The samples were gently rotated overnight at 4 °C and assessed as indicated above.

### GEF exchange assay

The GEF exchange assay was carried out using an *N*-MAR-GTP-based assay (BK100, Cytoskeleton) according to the manufacturer’s instructions. Two microliters of 15 μM Rheb-GDP or 7.5 μM RhoA and 2.5 μL of 0.3 mg/mL BSA were added to 7.5 μL of 2× exchange reaction buffer (40 mM Tris (pH 7.5), 100 mM NaCl, 20 mM MgCl_2_, and 1.5 μM *N*-MAR-GTP) in 384-well plates. The plates were examined immediately (excitation, 485 ± 20 nm; emission, 535 ± 25 nm) in a Spark™ 10 M multimode microplate reader (TECAN). After six readings (total 180 s), 3 μL of 50 μM EGFR or 2.5 μM human Dbs or ddH_2_O was added to the wells, and readings were immediately resumed (120 readings over a total of 60 min). For concentration gradients, 3 μL of 50 μM, 100 μM, or 150 μM EGFR was added to the wells and assessed as indicated above. To inhibit the GEF activity of EGFR, 5 mM erlotinib, afatinib, or BIEGi-1 was added to 50 μM EGFR, and then 3 μL of the mixture was added to the wells and subsequently assessed as indicated above.

### Structural analyses

The EGFR-TKD (amino acids, 712–979) and Rheb (amino acids, 1–184) complex was modeled using AlphaFold2-multimer^57^ available on the Google colaboratory server.^81^ Structures were analyzed and displayed using the PyMOL Molecular Graphics System, version 3.0.2., from Schrödinger.

### Multiple sequence alignment

The FASTA format of EGFR from the universal protein resource (http://www.uniprot.org)^82^ was used in multiple sequence alignment using EMBL-EBI (https://www.ebi.ac.uk/Tools/msa/clustalo/).^83^ JalView software was used to visualize the alignment.^84^

### Patient samples

This retrospective study was approved by the Institutional Ethics Committees at Guangdong Provincial People’s Hospital (approval number: KY2023-441-01) with exemption from obtaining informed consent. The tumor tissues were obtained from lung cancer patients who had EGFR-activating mutations and received either erlotinib or afatinib only as their neoadjuvant therapies.

### IHC

Paraffin-embedded tissue was sliced into 3-μm-thick transverse sections. Paraffin-embedded sections were gradually dewaxed by xylene and treated in an ethanol gradient to water. After heat-induced antigen retrieval, the sections were blocked with 3% hydrogen peroxide. The sections were incubated with primary antibodies at 4 °C overnight, followed by incubation with secondary antibody and DAB (Zsbio, ZLI-9017) detection. Hematoxylin counterstain was added for 10 s. In each step, the slides were washed vigorously with PBS three times for 5–10 min each. Primary antibodies included anti-AKT (pan) (C67E7) antibody (1:500, CST# 4691), anti-phospho-AKT (Ser473) (D9E) antibody (1:500, CST# 4060), anti-S6k1 antibody (1:50, abcam# ab32359), and anti-S6K1(phospho T389 + T412) antibody (1:50, abcam# ab60948). The stained tissue sections were imaged using a microscope imaging system (CellSens standard, Olympus BX53) at 20× and 40× magnification. Two pathologists independently evaluated the staining throughout the entire tissue section.

The immunoreactive score (IRS) for each antibody in each case was calculated by multiplying the score for the proportion of positive cells (P) by the staining intensity score (I): Q = P × I. P was assigned the score of 0 (no staining), 1 ( < 10% of neoplastic cell staining), 2 (10%–50% of neoplastic cell staining), 3 (51%–80% of neoplastic cell staining), or 4 (81%–100% of neoplastic cell staining). I was assigned the score of 1 (weak staining), 2 (moderate staining), or 3 (strong staining). The maximum score was 12. The two pathologists simultaneously evaluated and discussed any IRS that was scored differently.

For Fig. 1a, b, statistical analyses were conducted by calculating the ratio of phosphorylated to total protein for each sample, followed by statistical tests. All data were documented in Supplementary information, Table S1.

### Statistical analysis

Statistical analyses of the data were performed using Prism 9 (GraphPad) software. The experiments were performed multiple times to ensure reproducibility.

## Supplementary information

Supplementary information, Fig. S1

Supplementary information, Fig. S2

Supplementary information, Fig. S3

Supplementary information, Fig. S4

Supplementary information, Fig. S5

Supplementary information, Fig. S6

Supplementary information, Fig. S7

Supplementary information, Fig. S8

Supplementary information, Fig. S9

Supplementary information, Fig. S10

Supplementary information, Table S1

Supplementary information, Table S2

Supplementary information, Table S3
