# Supplementary material for: Lysosomal EGFR acts as a Rheb-GEF independent of its kinase activity to activate mTORC1
Source: Cell Res. 2025 Apr 21;35(7):497–509. doi: 10.1038/s41422-025-01110-x (PMC12205066; doi:10.1038/s41422-025-01110-x)
Supplement: Supplementary file 1 — Supplementary information, Fig. S1 [file 41422_2025_1110_MOESM1_ESM.pdf]

Supplementary Figure 1

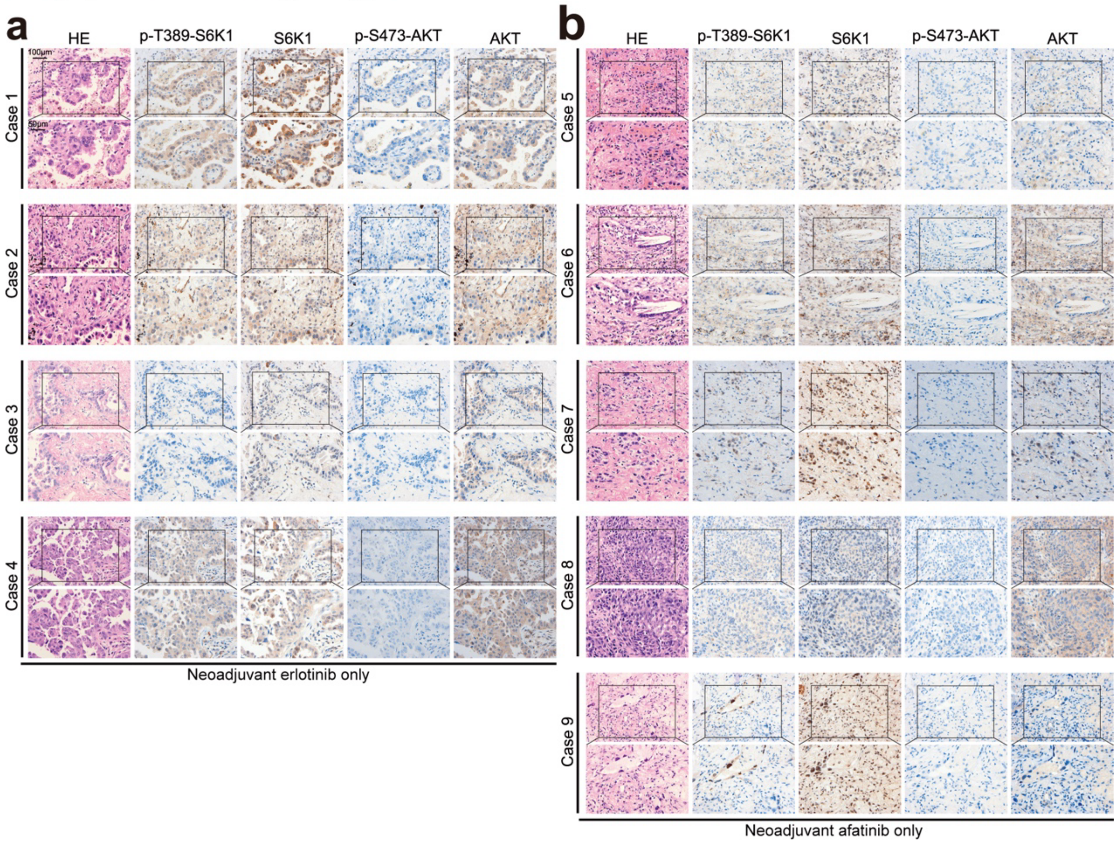

Supplementary Figure 1 Afatinib is more effective than erlotinib at impairing mTORC1 activation in lung adenocarcinoma patients harboring EGFR mutation.

(**a**) and (**b**) Immunohistochemical analysis of nine lung cancer patients. Representative immunohistochemical staining of p-T389-S6K1, S6K1, p-S473-AKT, and AKT using lung cancer tissues from the patients receiving neoadjuvant erlotinib (**a**) or afatinib (**b**).
