# Supplementary material for: Lysosomal EGFR acts as a Rheb-GEF independent of its kinase activity to activate mTORC1
Source: Cell Res. 2025 Apr 21;35(7):497–509. doi: 10.1038/s41422-025-01110-x (PMC12205066; doi:10.1038/s41422-025-01110-x)
Supplement: Supplementary file 2 — Supplementary information, Fig. S2 [file 41422_2025_1110_MOESM2_ESM.pdf]

Supplementary Figure 2

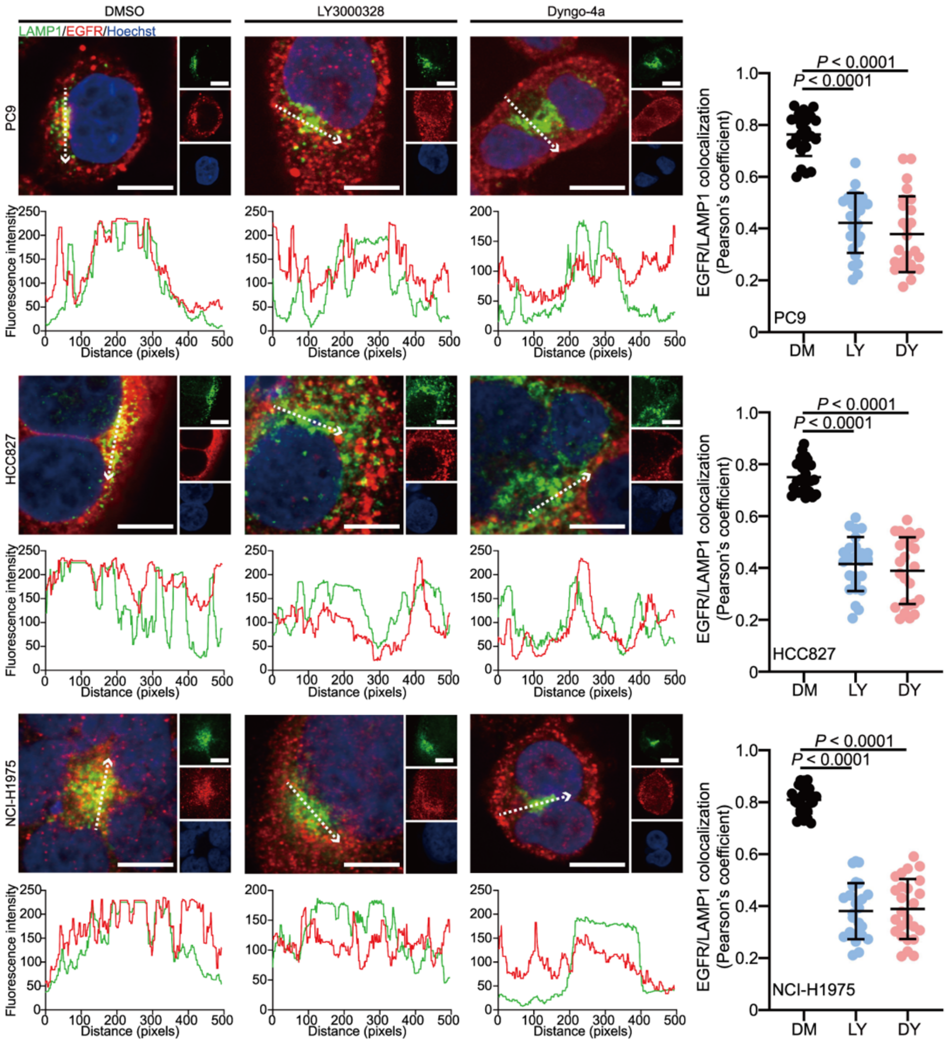

Supplementary Figure 2 LY3000328 or Dyngo-4a prevents the movement of endogenous mutant EGFR to the lysosome.

PC9, HCC827, or NCI-H1975 cells were treated with DMSO, 50 μM LY3000328 for 24 h, or 50 μM Dyngo-4a for 2 h, and analyzed by immunofluorescence of endogenous mutant EGFR and LAMP1. Representative images are shown. Scale bar, 10 μm. The arrow with a dashed line in each merged picture indicates the plane for generating line profiles of fluorescence intensities, as shown on the bottom. Quantification of EGFR/LAMP1 co-localization was performed on 25 individual cells sampled from three independent fields per condition. One-way ANOVA. Abbreviations: DM: DMSO; LY: LY300328; DY: Dyngo-4a.
