# Supplementary material for: Lysosomal EGFR acts as a Rheb-GEF independent of its kinase activity to activate mTORC1
Source: Cell Res. 2025 Apr 21;35(7):497–509. doi: 10.1038/s41422-025-01110-x (PMC12205066; doi:10.1038/s41422-025-01110-x)
Supplement: Supplementary file 3 — Supplementary information, Fig. S3 [file 41422_2025_1110_MOESM3_ESM.pdf]

Supplementary Figure 3

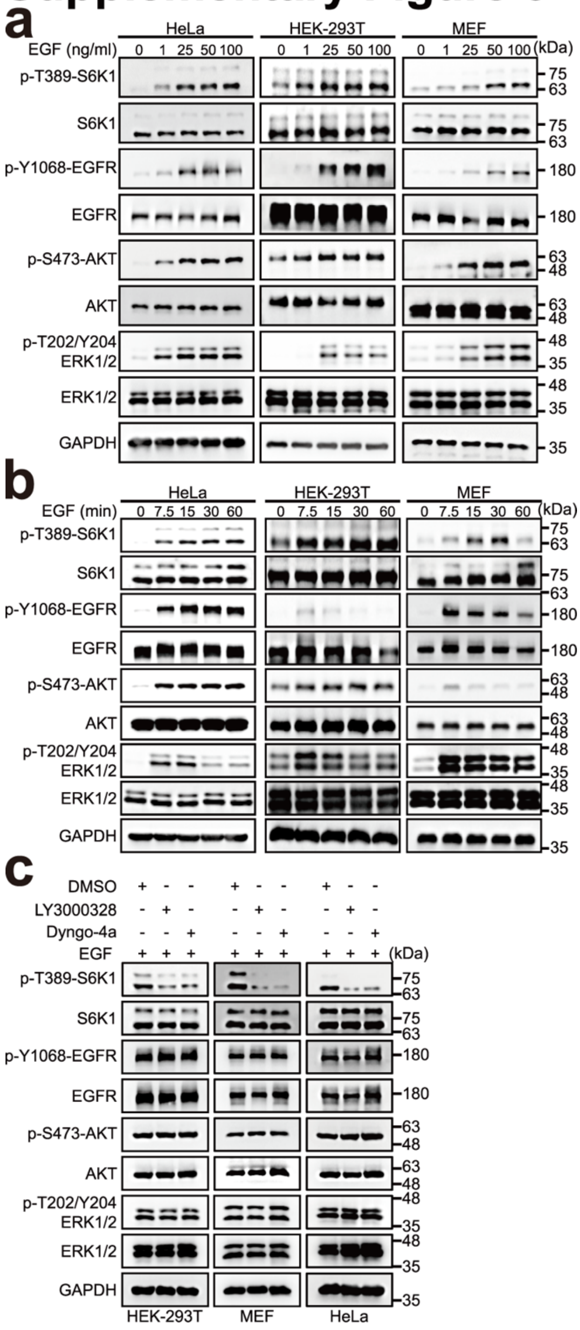

Supplementary Figure 3 mTORC1 activation is induced by EGF, and the peak is 100 ng/ml EGF for 30 minutes, which is inhibited by LY3000328 or Dyngo-4a.

(a) mTORC1 was activated by high-dose EGF stimulation. HeLa cells, HEK-293T cells, or MEFs were serum-starved for 24 h and stimulated with the indicated dose of EGF for 30 minutes. The levels of indicated proteins were probed by western blotting. **(b)** Full activation of mTORC1 was achieved around 30 minutes after EGF stimulation.

HeLa cells, HEK-293T cells, or MEFs were serum-starved for 24 h and stimulated with 100 ng/ml EGF. At 7.5, 15, 30 and 60 minutes, the levels of indicated proteins were probed by western blotting. **(c)** LY3000328 or Dyngo-4a impairs the activation of mTORC1 for WT EGFR cells upon EGF stimulation. HEK-293T, MEF, or HeLa cells were serum-starved, treated with DMSO, 50 μM LY3000328 for 24 h or 50 μM Dyngo-4a for 2 h, before stimulation with 100 ng/ml EGF for 30 minutes and analysis by western blotting.
