# Supplementary material for: Lysosomal EGFR acts as a Rheb-GEF independent of its kinase activity to activate mTORC1
Source: Cell Res. 2025 Apr 21;35(7):497–509. doi: 10.1038/s41422-025-01110-x (PMC12205066; doi:10.1038/s41422-025-01110-x)
Supplement: Supplementary file 4 — Supplementary information, Fig. S4 [file 41422_2025_1110_MOESM4_ESM.pdf]

Supplementary Figure 4

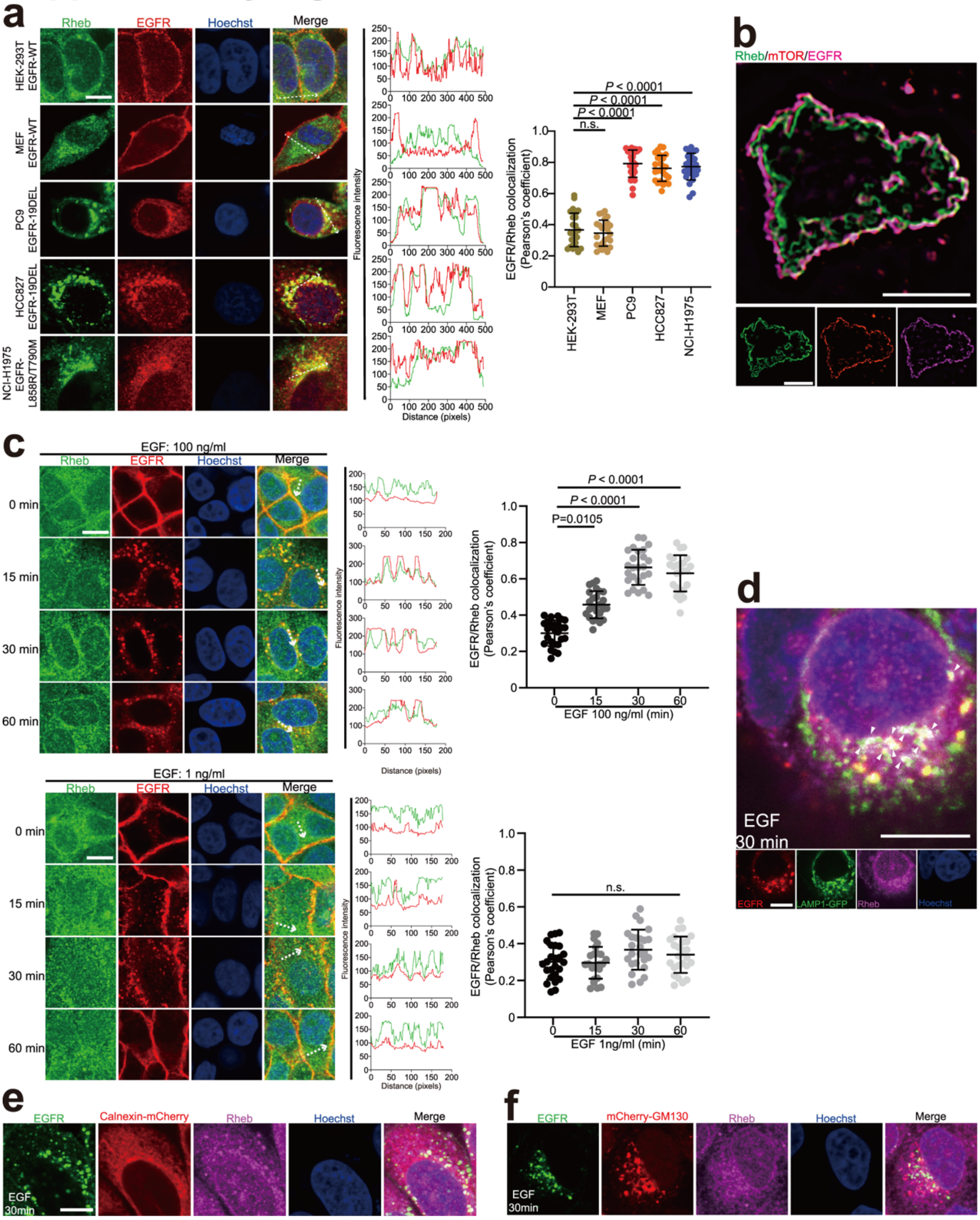

Supplementary Figure 4 Localization of EGFR and Rheb in mutant and WT EGFR-expressing cells treated with or without EGF.

(**a**) The oncogenic mutant EGFR and Rheb exhibited colocalization in the vicinity of the nucleus, but WT EGFR did not. The indicated cells were labelled with EGFR (red) and Rheb (green) and analyzed by immunofluorescence. Scale bar, 10 μm. The arrow

with a dashed line in each merged picture indicates the plane for generating line profiles of fluorescence intensities, as shown on the right. Quantification of EGFR/Rheb co-localization was performed on 25 individual cells sampled from 5 independent fields per condition. One-way ANOVA. (**b**) co-localization of endogenous mutant EGFR with Rheb and mTOR on lysosomal surface by Lyso-IP assay. Immunofluorescence of endogenous Rheb (green), EGFR (pink) and mTOR (red) on lysosomal surface using super-resolution structured illumination microscopy (SIM). Scale bar, 5 µm. (**c**) Endogenous WT EGFR co-localized with Rheb in cells treated with a high or low concentration of EGF for the indicated time. HeLa cells were serum-starved and then stimulated with a time course of EGF prior to immunofluorescent labeling of EGFR (red) and Rheb (green). Scale bar, 10 µm. The arrow with a dashed line in each merged picture indicates the plane for generating line profiles of fluorescence intensities, as shown on the right. Quantification of EGFR/Rheb co-localization was performed on 25 individual cells sampled from 3 independent fields per condition. One-way ANOVA. (**d**) EGFR colocalizes with Rheb on lysosomes in cells treated with EGF for 30 minutes. HeLa cells stably expressing LAMP1-GFP were serum-starved, stimulated with 100 ng/ml EGF for 30 minutes, and analyzed by immunofluorescence. Scale bar, 10 µm. (**e**) and **(f**) EGFR and Rheb did not co-localize with the endoplasmic reticulum and Golgi apparatus in cells treated with EGF for 30 minutes. HeLa cells stably expressing Calnexin-mCherry (endoplasmic reticulum marker) (**e**) or mCherry-GM130 (Golgi apparatus marker) (**f**) were serum-starved, stimulated with 100 ng/ml EGF for 30 minutes, and then analyzed by immunofluorescence. Scale bar, 10 µm.
