# Supplementary material for: Lysosomal EGFR acts as a Rheb-GEF independent of its kinase activity to activate mTORC1
Source: Cell Res. 2025 Apr 21;35(7):497–509. doi: 10.1038/s41422-025-01110-x (PMC12205066; doi:10.1038/s41422-025-01110-x)
Supplement: Supplementary file 5 — Supplementary information, Fig. S5 [file 41422_2025_1110_MOESM5_ESM.pdf]

Supplementary Figure 5

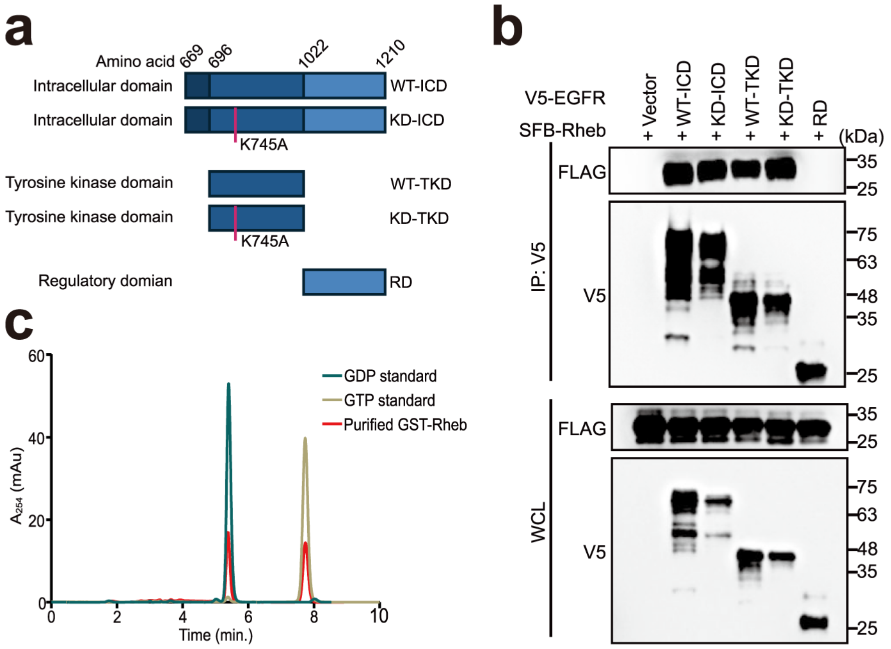

Supplementary Figure 5 EGFR-TKD interacts with Rheb.

(**a**) Schematic depiction of the intracellular domain of EGFR. (**b**) The EGFR-TKD binds Rheb. HEK-293T cells stably expressing SFB (S protein tag, FLAG tag, and Streptavidin binding peptide)-Rheb were transfected with the indicated V5-tagged plasmids and subjected to immunoprecipitation and analyzed by western blotting. (**c**) Purified GST-Rheb was bound to GDP and GTP in HPLC analysis.
