# Supplementary material for: Lysosomal EGFR acts as a Rheb-GEF independent of its kinase activity to activate mTORC1
Source: Cell Res. 2025 Apr 21;35(7):497–509. doi: 10.1038/s41422-025-01110-x (PMC12205066; doi:10.1038/s41422-025-01110-x)
Supplement: Supplementary file 6 — Supplementary information, Fig. S6 [file 41422_2025_1110_MOESM6_ESM.pdf]

Supplementary Figure 6

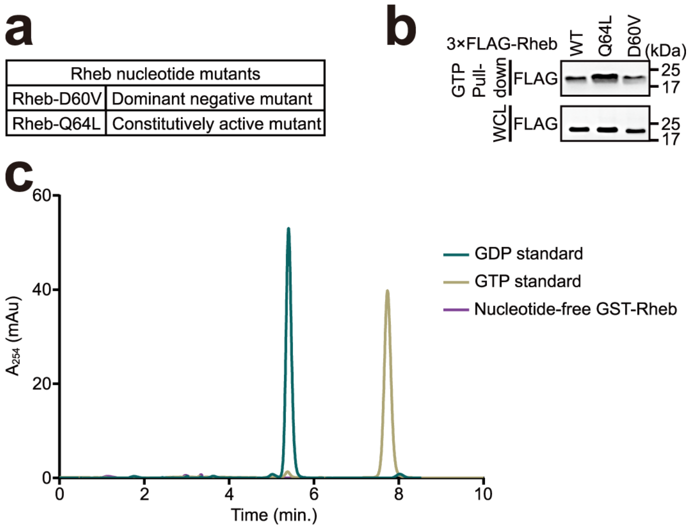

Supplementary Figure 6 functional characterization of Rheb variants and HPLC analysis of nucleotide-free Rheb.

(**a**) The table summarizes the two Rheb mutants used in this study. (**b**) HEK-293T cells transiently transfected with the indicated plasmids were lysed, subjected to GTP-agarose pulldown, and analyzed by western blotting. (**c**) Nucleotide-free GST-Rheb was identified by HPLC.
