# Supplementary material for: Lysosomal EGFR acts as a Rheb-GEF independent of its kinase activity to activate mTORC1
Source: Cell Res. 2025 Apr 21;35(7):497–509. doi: 10.1038/s41422-025-01110-x (PMC12205066; doi:10.1038/s41422-025-01110-x)
Supplement: Supplementary file 7 — Supplementary information, Fig. S7 [file 41422_2025_1110_MOESM7_ESM.pdf]

Supplementary Figure 7

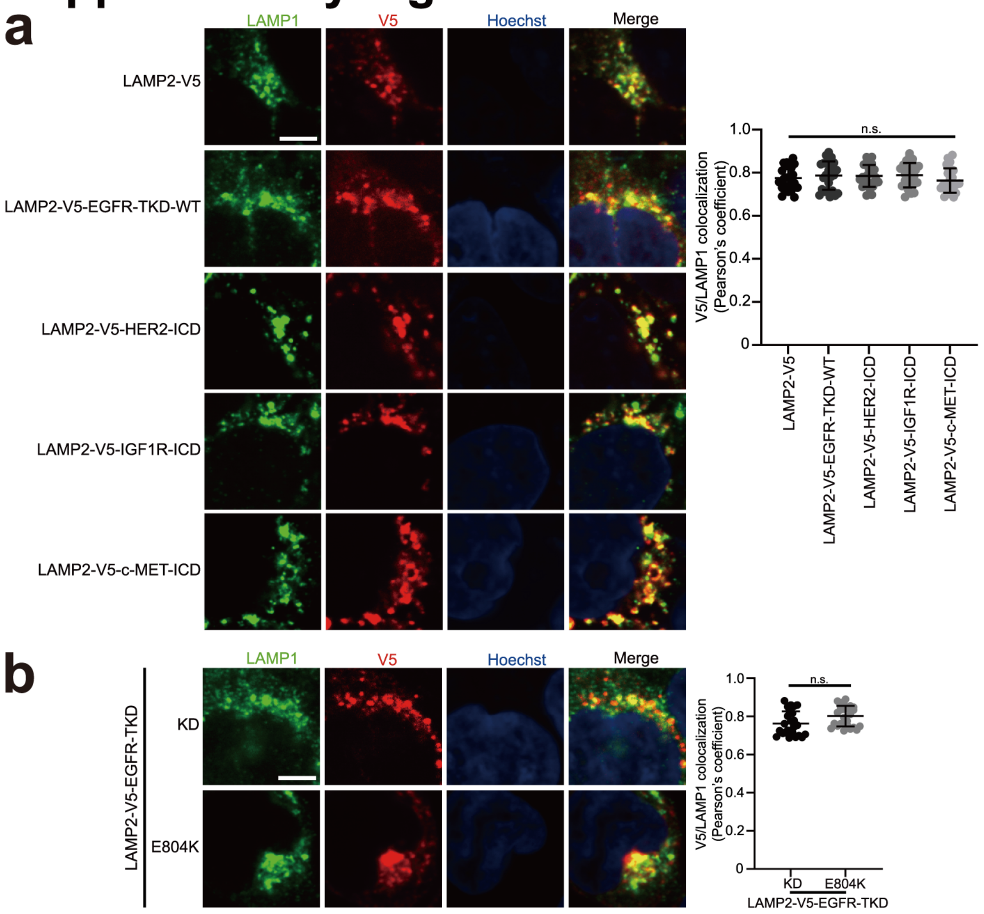

Supplementary Figure 7 The LAMP2-V5-RTKs-TKD/ICD are co-localized with LAMP1.

(**a**) HEK-293T cells stably expressing the indicated plasmids were serum-starved for 24 h, fixed, stained with antibodies against V5 and LAMP1, and then analyzed by immunofluorescence. Scale bar, 5 μm. Quantification of V5/LAMP1 co-localization was performed on 25 individual cells sampled from 3 independent fields per condition. One-way ANOVA. (**b**) HEK-293T cells stably expressing the indicated plasmids were serum-starved for 24 h, fixed, stained with antibodies against V5 and LAMP1, and then analyzed by immunofluorescence. Scale bar, 5 μm. Quantification of V5/LAMP1 co-localization was performed on 25 individual cells sampled from 3 independent fields per condition. Two-tailed unpaired t-test.
