# Supplementary material for: Lysosomal EGFR acts as a Rheb-GEF independent of its kinase activity to activate mTORC1
Source: Cell Res. 2025 Apr 21;35(7):497–509. doi: 10.1038/s41422-025-01110-x (PMC12205066; doi:10.1038/s41422-025-01110-x)
Supplement: Supplementary file 8 — Supplementary information, Fig. S8 [file 41422_2025_1110_MOESM8_ESM.pdf]

Supplementary Figure 8

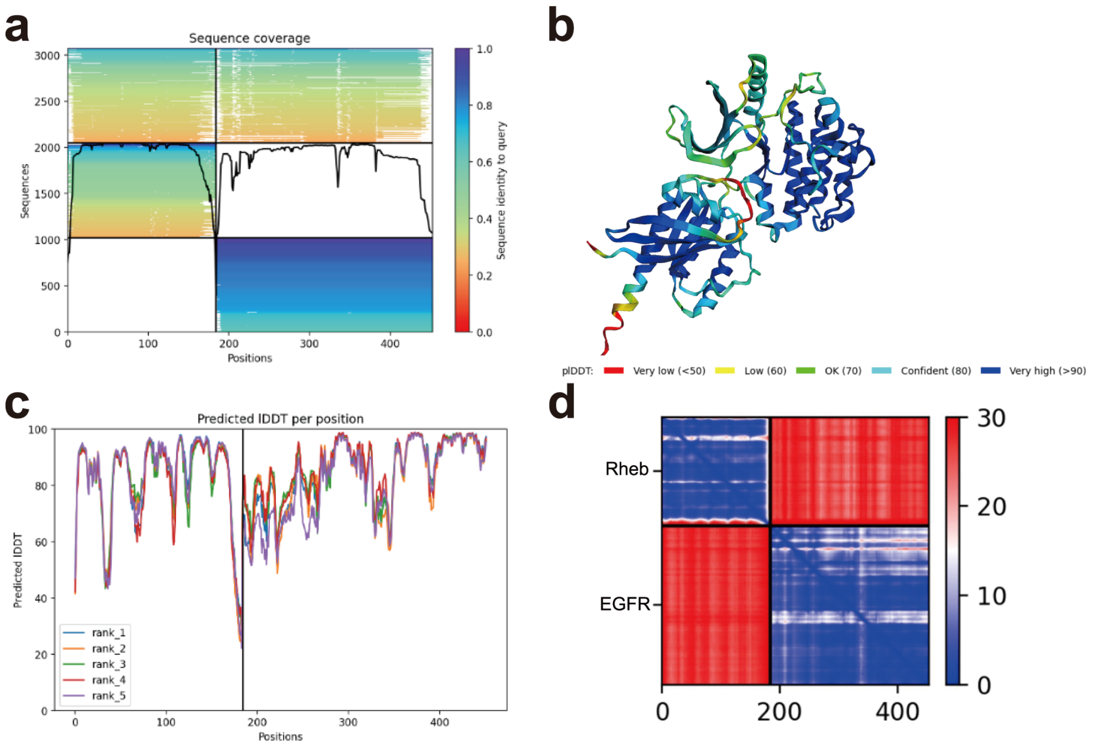

Supplementary Figure 8 The quality of predicted EGFR-Rheb complex by AlphaFold2-Multimer.

(**a**) and (**b**) The structure shown in **Fig. 4a** is colored by pLDDT value, a measure of the confidence that an amino acid is positioned correctly relative to neighboring residues. (**c**) pLDDT values for the EGFR-Rheb complex displayed as a graph. pLDDT values for each model are shown as differently colored lines. The values for rank_1 to rank_5 are 83.8, 83.8, 84.4, 84.2, and 82.2, respectively. The highest value of rank_3 was used in the main figure. (**d**) Predicted alignment error plot for the EGFR-TKD and Rheb interaction.
