# Supplementary material for: Lysosomal EGFR acts as a Rheb-GEF independent of its kinase activity to activate mTORC1
Source: Cell Res. 2025 Apr 21;35(7):497–509. doi: 10.1038/s41422-025-01110-x (PMC12205066; doi:10.1038/s41422-025-01110-x)
Supplement: Supplementary file 9 — Supplementary information, Fig. S9 [file 41422_2025_1110_MOESM9_ESM.pdf]

Supplementary Figure 9

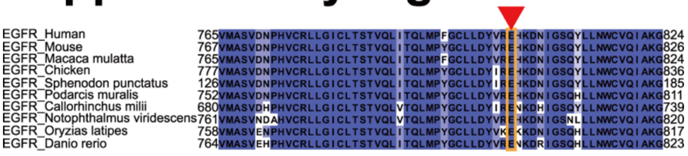

Supplementary Figure 9 Multiple sequence alignment of EGFR in vertebrates.

The glutamate residue is a conserved residue in vertebrates.
