# Supplementary material for: Lysosomal EGFR acts as a Rheb-GEF independent of its kinase activity to activate mTORC1
Source: Cell Res. 2025 Apr 21;35(7):497–509. doi: 10.1038/s41422-025-01110-x (PMC12205066; doi:10.1038/s41422-025-01110-x)
Supplement: Supplementary file 10 — Supplementary information, Fig. S10 [file 41422_2025_1110_MOESM10_ESM.pdf]

Supplementary Figure 10

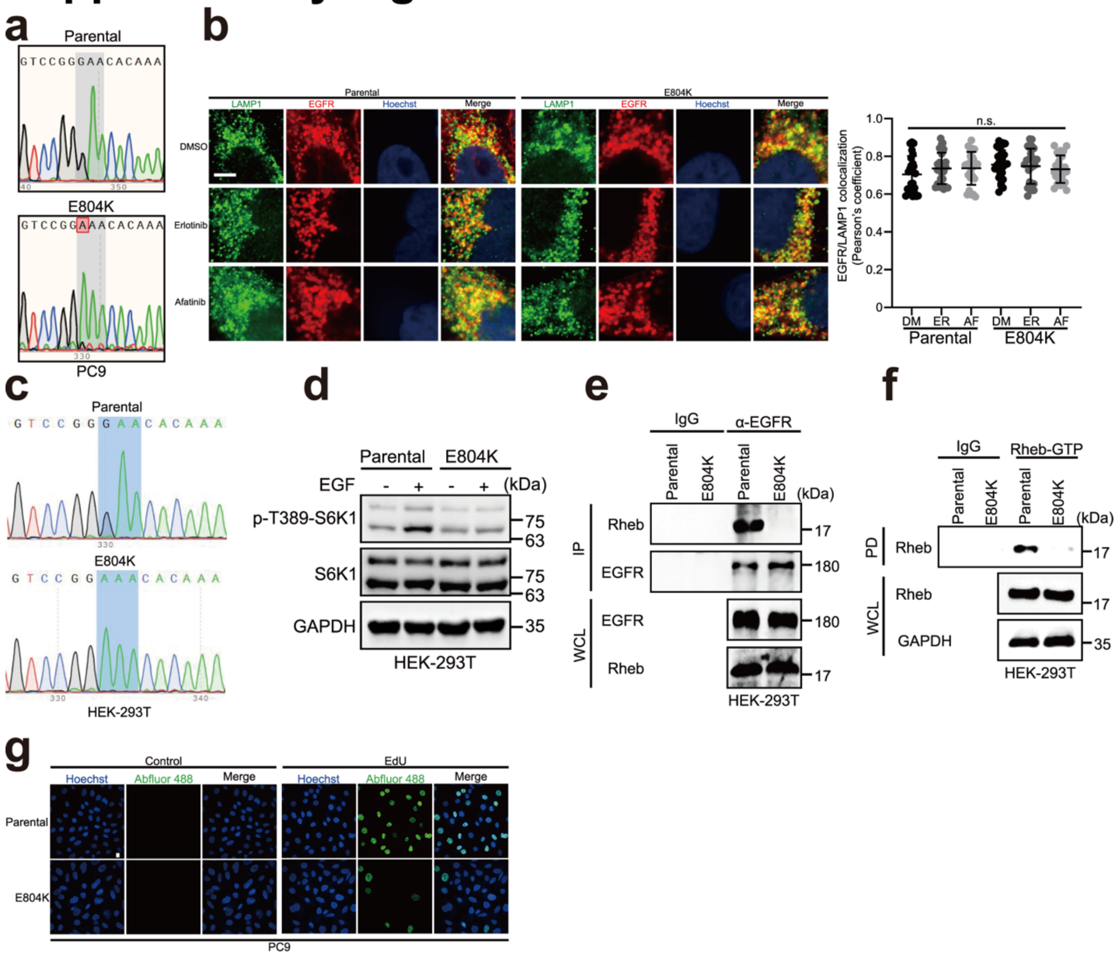

Supplementary Figure 10 Aberrant Rheb-GEF activity of EGFR impairs mTORC1 activation and cell growth.

(**a**) Generation of EGFR-E804K knock-in was confirmed for PC9 cells by Sanger sequencing. (**b**) EGFR was observed to co-localize with LAMP1 in both parental and EGFR-E804K knock-in PC9 cells, regardless of treatment with erlotinib or afatinib. Cells were treated with 25 nM of either erlotinib or afatinib for 12 h, subsequently stained for EGFR (red) and LAMP1 (green), and analyzed by immunofluorescence microscopy. Scale bar, 1 µm. Quantification of EGFR/LAMP1 co-localization was performed on 25 individual cells sampled from 3 independent fields per condition. Abbreviations: DM: DMSO; ER: erlotinib; AF: afatinib. (**c**) Generation of EGFR-E804K knock-in was confirmed for HEK-293T cells by Sanger sequencing. (**d**) mTORC1 activation was inhibited upon EGF stimulation in EGFR-E804K knock-in

cells. EGFR-E804K knock-in HEK-293T cells were serum-starved for 24 h, stimulated with or without 100 ng/ml EGF for 30 minutes, and analyzed by western blotting. (**e**) The EGFR-Rheb interaction was disrupted in EGFR-E804K knock-in cells. EGFR-E804K knock-in HEK-293T cell lysates were subjected to immunoprecipitation using IgG or anti-EGFR antibody and analyzed by western blotting. (**f**) The level of GTP-bound Rheb was decreased in EGFR-E804K knock-in cells. EGFR-E804K knock-in HEK-293T cell lysates were subjected to immunoprecipitation using Rheb-GTP agarose and analyzed by western blotting. (**g**) The proliferation of parental and E804K knock-in cells detected by EdU assay. Scale bar, 10 µm.
